# Deregulation of m6A-RNA methylation impairs adaptive hypertrophic response and drives maladaptation via mTORC1-S6K1-hyperactivation and autophagy impairment

**DOI:** 10.1186/s12964-025-02509-0

**Published:** 2025-12-02

**Authors:** Karthika Annamalai, Soniya Dilliker, Eric Buchholz, Ricardo Castro-Hernández, Nikita Panyam, Alessa Pommeranz, Pascal Wiederhake, Nelly Wery von Limont, Nina Hempel, Verena Ebner, Surabhi Swarnkar, Belal A. Mohamed, Katrin Streckfuss-Bömeke, Sabine Steffens, Stephan Herzig, Antje Ebert, Andre Fischer, Karl Toischer

**Affiliations:** 1 Department of Cardiology and Pneumology, University Medical Center Göttingen (UMG), Göttingen, Germany; 2 DZHK (German Centre for Cardiovascular Research) Partner Site Göttingen, Göttingen, Germany; 3 DZHK (German Centre for Cardiovascular Research) Partner Site Munich Heart Alliance, Munich, Germany; 4 Department for Epigenetics and Systems Medicine in Neurodegenerative Diseases, German Center for Neurodegenerative Diseases (DZNE), Göttingen, Germany; 5 Cluster of Excellence MBExC, University of Göttingen & University Medical Center Göttingen, Göttingen, Germany; 6 Institute for Pharmacology and Toxicology of the University of Würzburg, Würzburg, Germany; 7 Institute for Diabetes and Cancer (IDC), Helmholtz Diabetes Center (HDC), Helmholtz Zentrum München (Helmholtz Munich), Munich, Germany; 8 Institute for Cardiovascular Prevention (IPEK), University Hospital, LMU Munich, Munich, Germany; 9 Clinic for Cardiology and Pneumology, University Hospital Göttingen, Robert-Koch-Strasse 40, Göttingen, 37075 Germany; 10 Medical Clinic I, Cardiology and Angiology, Justus Liebig University, Giessen, Germany

**Keywords:** Pathological remodeling, N6-methyladenosine, Mouse, Cardiac hypertrophy, Atrophy, Apoptosis

## Abstract

**Background:**

Pressure overload first leads to compensated hypertrophy and secondary to heart failure. m6A-RNA methylation is a fast process for the adaptation of cell composition. m6A-RNA-methylation is regulated by the demethylase, fat mass and obesity-associated protein* (*FTO), and FTO protein levels are diminished in heart failure. Cardiomyocyte-specific FTO-transgenic/knockout-mice have shown the relevance of FTO in pressure overload remodeling. However, its functional downstream regulatory mechanisms are still unclear. In this study, we discover the harmful signaling pathways that are triggered by m6A imbalance and FTO loss, which eventually lead to adverse cardiac remodeling and heart failure.

**Methods:**

FTOcKO animals were generated by crossing FTO^fl/fl^ mice with $\alpha$-*MHC* Cre mice using Cre-lox system. Control and the FTOcKO animals groups were subjected to TAC (transverse aortic constriction) surgery. Echocardiography was performed 1-week post-TAC surgery. MeRIP (m6A RNA immunoprecipitation) sequencing was performed from the heart tissues of mice after one week TAC surgery. Additionally, the mechanistical interrelation between the signaling pathways during FTO loss and adverse cardiac remodeling were investigated in human iPS-CMs (hiPS-CMs).

**Results:**

One week post-TAC surgery, FTOcKO mice showed impaired cardiac function (EF: CreC TAC (45%) vs. FTOcKO TAC (25%), *p* < 0.0001) and increased LVID (CreC TAC(3.9 mm) vs. FTOcKO TAC (4.8 mm), *p* < 0.0001), indicating a lack of adaption to pressure overload. Knockdown of FTO in hiPS-cardiomyocytes also reduced endothelin-induced hypertrophic response.

MeRIP-seq data of FTOcKO mice showed that the differentially hypermethylated transcripts were associated with cardiac apoptosis inhibition (CDK1, CFLAR), mTORC1 signaling pathway (AKT1S1) and autophagy regulation (TFEB). mTORC1 was identified as a central player of dysregulation with hyperactivation of its canonical substrates phospho-S6K1 (Thr 389) and phospho-S6 (ser235/236) ex-vivo (FTOcKO) and in-vitro (FTO-KD-hiPS-CMs).

Moreover, FTO-deficient cardiomyocytes cause autophagic flux impairment and defective autophagy. The effect of atrophy and induced apoptosis upon FTO-m6A imbalance could be rescued by pharmacological inhibiton of the mTORC1-S6K1 pathway.

**Conclusions:**

Downregulation of FTO leads to mTORC1-S6K1 hyperactivation that shift the compensative hypertrophic response to atrophy and apoptosis leading to progressive heart failure. These findings might pave the way for the development of novel therapeutic targets for the early phases of heart failure treatments.

**Supplementary Information:**

The online version contains supplementary material available at 10.1186/s12964-025-02509-0.

## Background

Cardiac hypertrophy is a well-characterised adaptive response, which increases individual cell size through enhanced protein synthesis and enlarged sarcomere organisation [1, 2]. Under consistent pathological stimuli the initial adaptive cardiac hypertrophy marked as compensatory process, transits to maladaptive pathological remodelling with increased LV dilatation and reduced ejection fraction. Over the last few decades, several publications propose various signaling and molecular events including impaired protein quality, metabolic reprogramming, reinduction of fetal gene programming, immunomodulation or aberrant Ca2 + handling involved in the transition of pathological hypertrophy [3, 4]. Notably, posttranscriptional modifications are identified as the new class of regulators of protein expression, that govern several signaling pathways [5]. Therefore, extensive research is highly demanded to delineate the critical posttranscriptional modifications and the signaling mechanisms that regulate the development of pathological hypertrophy and heart failure.

N6-methyladenosine (m6A) is identified as the most abundant internal postranscriptional RNA modification, which is dynamic and reversible and regulates gene expression in different cell types including myocardium [6–8]. The m6A modification occurs in a consensus motif “DRm6ACH” (D = A,U or G; R = A,G; H = A,C,or U) [9, 10]. The enzymes that catalyze m6A methylations are known as methyl transferases (METTL3, METTL14, WTAP, METTL16) and demethylases (FTO, ALKBH5) [11]. The m6A binding proteins (a.k.a. m6A readers) that belong to YTH protein family, affect the mRNA-transcript fate by altering/modulating translation, cleavage and degradation processes [12]. In contrast to the classical transcriptional regulation, m6A-RNA methylation can modify protein expression independent of transcription [13–15].

Recent studies from us and other groups highlighted the significance of m6A RNA methylation in maintaining cardiac homeostasis, cardiac hypertrophy and heart failure [5, 14, 16, 17]. Especially in early disease states like compensated hypertrophy in the heart [14] or also in early neurodegenerative diseases [18], the regulation of m6A methylation is more prominent, indicating a fast adaption system to external stimuli of the cells. In addition, FTO is downregulated in the failing hearts of humans and rodents; and induced FTO overexpression mitigates cardiac dysfunction indicating the essential role of FTO in cardiac function [19, 20]. Consistently, our previous study in cardiomyocyte specific FTO knockout (FTOcKO) animals showed that the loss of FTO causes maladaptive cardiac remodeling without compensatory hypertrophy in response to 4 weeks of PO (TAC with 27G needle) [14], however, its downstream functional regulatory mechanisms are not yet known.

mTORC1 is the master regulator of cell growth and metabolism which functions in response to nutrients and growth stimuli. It has been demonstrated that mTORC1 is required for the cardiac adaptation to pressure overload since its loss impairs compensatory hypertrophy in response to pressure overload, leading to fast ventricular dilatation, apoptosis, and mitochondrial derangements [21–23]. However, there is evidence that mTORC1 signaling alone not sufficient for compensatory hypertrophy and it works together with other signaling pathways in response to hypertrophic stimuli in the heart. Although the involvement of mTORC1 in compensatory hypertrophy is still debatable [21], in this study, we made efforts to identify the interrelation between changes in FTO dependent m6A methylations, mTORC1 and autophagy, which works as signaling events of cardiac hypertrophy and remodeling.

This novel functional regulations of FTO add a new dimension of therapeutic interventions for the treatment of cardiac hypertrophy and heart failure at the early phase.

## Methods

The detailed description of the Methods is in the Supplementary file METHODS S1.

### Mice

For the animal experiments, all the experimental investigations conform to the Guide for the Care and Use of Laboratory animals (NIH publication No.85–23, revised 1996) and was implemented in accordance with the ethical standards laid down in the Declaration of Helsinki 1964. Cardiomyocyte specific FTO knockout (FTOcKO) mice were generated by mating male FTO^fl/fl^ mice (Exon 3 of *Fto* is flanked by loxP sites, was available inhouse) with the cardiomyocyte specific *αMHC-Cre* females (*αMHC-Cre*, Jackson no. 011038, C57BL/6N and C57BL/6 J mixed background [Agah et al. 1997]). The αMHC-Cre line expresses a constitutive (non-inducible) Cre recombinase under the control of the αMHC promoter. And for the experiments, *αMHC-Cre*^+^;*FTO* ^fl/fl^ mice (Cre + fl/fl) bearing the homozygous *Fto* knock-out were used and named as FTOcKO mice. Further *αMHC-Cre*^+^ mice (Cre + wt/wt) were used as experimental control to rule out Cre- recombinase and loxP system derived effects and named as CreC mice.

### Transverse aortic constriction (TAC)

TAC surgery was carried with minimally invasive approach. 8-week-old mice were randomly selected for both Sham and TAC surgery. Initially, the animals were anaesthetized using intraperitoneal injections with mixture of Medetomidin (0.5 mg/kg), Midazolam (5 mg/kg) and Fentanyl (0.05 mg/kg). A 26 gauge needle was tied against the aorta using a 5–0 Polyviolene (non-absorbable) suture for TAC surgery, and Sham animals underwent the similar procedure without banding of the transverse aorta.

### Echocardiography

Transthoracic echocardiography using Vevo 2100 system (Visualsonics) were examined for mice that underwent TAC or Sham surgery (1 week post-surgery). The animals were anaesthetized using 1.5% isoflurane, and the heart rate, respiratory rate and body temperature were monitored. 2D guided M-mode images were recorded in both long axis and short axis with the help of of an MS-400 30 MHz transducer (Visualsonics) (Pistner et al. 2010). For the analysis of the recorded images, VevoLab software were applied (Version 3.1.0, Visualsonics). The examiner were blinded for the assigned animal groups.

### Murine LV isolation

For the heart isolation, the mice were anesthetized with isoflurane and sacrificed by cervical dislocation. The thorax were opened and the exposed heart were then cut above the aorta and washed in sterile NaCl solution. Further, the heart was perfused with sterile NaCl by inserting a 21 gauge blunt needle into the aorta. The heart was weighed, and the LV snap alone were separated and frozen in liquid nitrogen for the experimental investigations. The tibia was also isolated from the muscle tissue and its length was measured in order to calculate heart weight/tibia length ratio.

### Methylated RNA immunoprecipitation (MeRIP)

MeRIP was performed by firstly isolating total RNA from the left ventricles of mice, and further purifying the mRNA by DNAse treatment and rRNA Depletion (NEB, #E6310, Ipswich, MA). The RNA was further fragmented and immunoprecipitated with anti-m6A polyclonal antibody (synaptic systems, #202,003). The eluted RNA was processed for cDNA library preparation using TruSeq stranded Total RNA Library Prep Kit (#20,020,596, Illumina) and sequenced with Illumina HiSeq2000. MeTpeak package was used for the detection of m6A enrichment in comparison to the input samples.

### Methylated RNA immunoprecipitation (MeRIP)- qPCR

For the validation of MeRIP-seq data, the m6A enrichment of the transcripts AKT1S1 and TFEB was analysed by performing MeRIP from the Scr and FTO silenced hiPS-CMs using Magna-MeRIP™ m6A Kit (catalog no. 17–10,499). MeRIP was carried as per the manual`s instruction. The eluted samples of m6A immunoprecipitation and IgG (control) RIP, were subjected to cDNA synthesis using Quantitect Reverse Transcription Kit Catalog no#205,313.

Further, cDNAs of m6A RIP and IgG RIP are subjected to real time PCR using the specific taqman pre-mixed primers for AKT1S1 (TaqMan Gene expression Assay ID Hs00982883_m1; AKT1S1); TFEB** (**TaqManTM Gene Expression Assay Hs00292981_m1; TFEB) from Invitrogen. The analysis for m6A enrichment is performed by using Δ ΔCt method, where the ΔCt for m6A and the negative control (IgG) is normalized with their respective inputs and further Δ ΔCt is obtained by subtracting by ΔCt of m6A with ΔCt of IgG (negative). And the fold enrichment is calculated with 2^(- Δ ΔCt). The fold enrichment is plotted in the graph for each transcript.

### Real time PCR

The total RNA extracted from Scr and siFTO hiPS-CMs were subjected to cDNA synthesis using Quantitect Reverse Transcription Kit (Cat. No. #205,313) and the corresponding cDNAs were considered for Real time PCR. The Taqman Universal PCR master mix (Cat. No. 4304437) and the specific qPCR primers for AKT1S1 (TaqMan Gene expression Assay ID Hs00982883_m1; AKT1S1); TFEB** (**TaqManTM Gene Expression Assay Hs00292981_m1; TFEB); and FTO (Taqman Gene expression Assay ID Hs01057143_m1; FTO) were purchased from Invitrogen.

### Human induced pluripotent stemcells (hiPSCs)

For this sutdy, the human patient derived pluripotent stemcells (hiPSCs) were kindly provided by Dr. Katrin Streckfuß-Bömeke (Institue for pharmacology and toxicology, University of Würzburg, Germany), PD Dr. Antje Ebert (University Medical Center Göttingen, Department of Cardiology and Pneumology) and by the core facility induced pluripotent stem cells (CF-iPSC), Helmholtz Munich. The hiPSCs were generated from patients with non-failing hearts.

### hiPS cells differentiation into cardiomyocytes (hiPS-CMs) and culturing

The hiPSCs were differentiated into beating ventricular cardiomyocytes with an adapted protocol from Kleinsorge and Cyganek et al. [24].

### siRNAs, hypertrophy inducer and inhibitors

For transfection experiments, siRNAs were purchased from Qiagen. For induction of hypertrophic responses in hiPS-CMs, Endothelin-1 (ET-1, Sigma #E7764) was used. For mTORC1-S6K1 inhibition, the inhibitors Rapamycin (#553,210-10MG) and PF-4708671 (#559,273-10MG) were used. For late-autophagy inhibition, the inhibitors bafilomycin A1 (CAS 88899–55.2) and chloroquine were used.

### Transfections in hiPS-CMs

The hiPS-CMs cultured in the 6 well plates/12 well plates were transfected with siRNAs using Hiperfect Transfection reagent (Qiagen; #301,705). 50 nM and 100 nM siRNAs for 12 well and 6 well plates respectively were considered for the transfection in hiPS-CMs.

### Antibodies for western blot and immunofluorescence (IF)

The antibodies used for western blot are all diluted in the ratio 1:1000 except for the house keeping genes (1:10,000). The order details of the antibodies are ANP(#ab126149), MYH7 (MA5-32,986), FTO (#NBP2-29,512), cleaved Caspase3 (#9664S), cleaved PARP (#9544S,#9541S), P-S6 Ribosomal protein (S235/236) (#4858S), P-p70 S6 kinase (T389) (#9234S), P-4E-BP1(T37/46) (#2855S), total S6 Ribosomal protein (#2217S), p70-S6Kinase(2708S), 4E-BP1(#9644 T), SQSTM1/p62 (#5114S), LC3B(#2775S), Actin(#4967) and GAPDH (#2118S), PRAS40 (#2610S), TFEB (#4240S), METTL3(#96391S), ALKBH5(#80283S), p-AKT(s473) (#9271S), AKT (#9272S).

For immunofluroscence the antibodies were diluted in the ratio 1:100: alpha-actinin(sarcomeric) (#A7732), phospho-S6(ser235,ser236) (#MA5-15,140), p62 (#GP62-C-WBC), LC3B (#2775S), anti-mouse IgG secondary antibody Alexafluor plus488 (#A32723), anti-rabbit IgG(H + L) cross adsorbed secondary antibody Alexafluor 555 (#10,082,602), FITC-affinipure anti-rabbit IgG (H + L) (#111–095-003), Cy3- conjugated affinipure anti-Guinea Pig IgG (H + L) (#706–165-148).

### Histology

Hearts were harvested from the mice and were fixed in 4% formalin, further embedded with paraffin and sectioned (6 µm). Those LV sections were considered for Immunohistochemistry and Immunofluorescence.

The sections were stained with fluorescein-conjugated wheat germ agglutinin (WGA-Alexa Fluor 594; Invitrogen) for the assessment of cross-sectional area using ImageJ software (NIH; Bethesda).

Further, the LV sections were stained for Fibrosis by using Picro Sirius Red Stain Kit (Connective Tissue Stain) #ab150681; and the fibrotic area is measured using ImageJ software NIH; Bethesda). And the % fibrosis is plotted in prism graph.

### TUNEL assay (for LV sections and for hiPS-CMs)

The LV sections were de-paraffinized, permeablized and the slides were labelled with TUNEL working solution (In situ Cell Death Kit, TMR red #12,156,792,910) or TUNEL Staining (#11,684,795,910—In Situ Cell Death Detection Kit, Fluorescein-Roche) and incubated at 37 °C for 1 h. The sections were finally washed with PBS before mounting with DAPI and proceeded with fluorescent microscopy analysis.

The TUNEL assay was performed in the hiPS-CMs by initially coating the cardiomyocytes in the coverslips (which were plated in the 12 well plates for the transfection and treatments).

### P-S6 staining (for LV sections and for hiPS-CMs)

Phospho-S6 staining was performed for the LV sections using the enhancer Tyramide Signal Amplication (TSA) kit (Alexa Fluor 488 Tyramide Super Bosst Kit # B40922).

Phospho-S6 staining were performed in hiPS-CMs by plating the cells in coverslips of 12 well plates (transfections were performed in 12 well plates). The fixed and permealized cells were incubated with primary antibodies (alpha-actinin and phospho-S6(ser235,ser236)) overnight at 4 °C. The following day, the cells were incubated with their respective secondary antibody solutions at RT for 1 h. The cells were mounted with DAPI for microscopic analysis.

### P62 and LC3 localization (for hiPS-CMs)

The CMs were double stained (as mentioned in 13.2.) Here, the cells were incubated with primary antibodies (SQSTM1/p62 and LC3 B) overnight at 4 °C. And the following day, the cells were washed and incubated with their respective secondary antibodies (FITC anti-rabbit and Cy3 anti-GP as mentioned in Sect. 10) and the stained coverslips were mounted with DAPI and headed for microscopic analysis.

### Tandem mRFP-GFP-LC3 assay (for hiPS-CMs)

The CMs were plated in 24 well plates, the cells were initially transduced with tandem sensor RFP-GFP-LC3B (catalog no. P36239), and transfected with siRNAs for scr and FTO, and subsequent treatment with or without Bafilomycin. The live imaging is performed for microscopical analysis.

### Dot blot assay for m6A enrichment (for hiPS-CMs)

The total mRNA of Scr and siFTO-hiPS-CMs, were initially denatured at 95 °C for 5 min to disrupt the secondary structure. Further, chilled on ice immediately for 5 min and spotted on activated PVDF membrane. The crosslinking of mRNA to the membrane is carried by UV light exposure for 5 min. Further, the membrane is blocked and incubated with primary antibody buffer overnight. The m6A antibody is purchased from Cell Signaling technology with cat. No. 56593S. The m6A spots are developed on the next day after incubating with corresponding secondary antibody solution.

### Statistical analysis

Statistical analysis of the echocardiographic data was performed in the GraphPad Prism Software (v. 8.4.2). Ordinary two-way ANOVA and Tukey`s multiple comparison analysis was performed for Echocardiographic analysis, WGA staining, Fibrosis and Densitometry analysis for mice groups (Sham/TAC and FTOcKO/Cre Control). Densitometry analysis of the protein bands of western blots for scr and siFTO-hiPS-CMs were examined by 1-sample t-tests (using GraphPad prism 9.5.1). The Results were normalized to their respective control conditions and the ratios in which a normal distribution of results cannot be proven, were analyzed. P value < 0.05 was considered as statistically significant.

## Results

### Cardiomyocyte-specific FTO ablation causes early dilatation and maladaptive cardiac phenotype

**Fig. 1:**
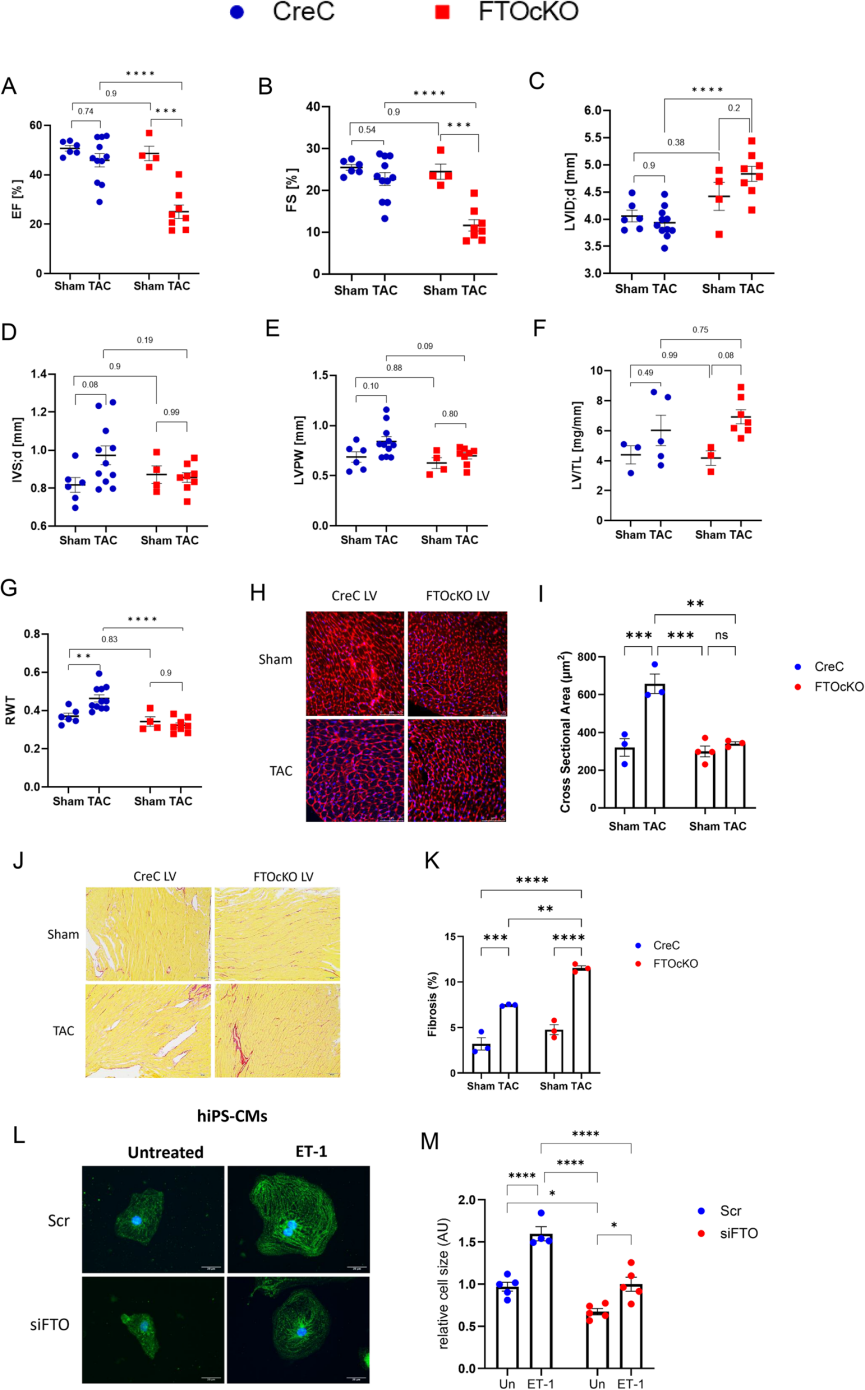
Cardiomyocyte specific FTO deficiency causes early dilatation and reduces compensatory hypertrophy. Two-month-old FTOcKO and Cre control animals were analyzed one week after TAC surgery using 26-G needle **A**-**I**. The Echocardiography is measured for each group: Cre control in blue (sham & TAC) and FTOcKO in red (sham and TAC); (**A**) The Ejection fraction (EF) and (**B**) Fractional Shortening (FS) which measures the cardiac function are presented in percentage. **C** LVID (left ventricular interdimensional diameter) is measured in mm; (**D**) Intraventricular septum thickness is measured in mm; (**E**) The LV posterior wall thickness (LVPW) is measured in mm; (**F**) The ratio of LV weight-to- tibia length (LV/TL in mg/mm) is calculated for each mice; (**G**) The relative wall thickness (RWT) is calculated by dividing the sum of anterior wall thickness (LVAW) and posterior wall thickness (LVPW) by the LV inner dimension (LVID) ((LVAW + LVPW)/LVID); (**H**) representative transverse sections of FTOcKO and Control mice stained using wheat germ agglutinin (WGA); (**I**) The cross-sectional area for the WGA stained sections are represented in µm^2^; (**J**) representative transverse sections of FTOcKO and Control mice stained using picro sirius stain kit; (**K**) the fibrotic area is represented in percentage; Each dot represents the measurement of one animal, significance levels presented using ordinary two-way ANOVA with Tukey’s correction for multiple comparison analysis. The mean with SEM is presented; mice n for A-G (CreC Sham, *n =* 3–6; CreC TAC, *n =* 5–11; FTOcKO Sham, *n =* 3–4; FTOcKO TAC, *n =* 7–8); mice n for I (CreC Sham, *n =* 3; CreC TAC, *n =* 3; FTOcKO Sham, *n =* 4; FTOcKO TAC, *n =* 3); mice n for K (CreC sham, *n =* 3; CreC TAC, *n =* 4; FTOcKO Sham, *n =* 3; FTOcKO TAC, *n =* 3). For hypertrophic analysis in vitro, hiPS-CMs were transfected with scr siRNAs (scr) or with FTO siRNAs (siFTO) and their hypertrophic response were studied by treating the silenced CMs with 3 nM of ET-1 (Endothelin-1) for 24 h; (**L**) show the representative images of scr and siFTO transfected CMs with or without ET-1. Green fluorescence represents the alpha-actinin (sarcomeric) and blue is stained with DAPI; (**M**) represents the hypertrophic measurement and the relative cell size for each CM is measured using ImageJ. The mean with SEM is presented. n represents cells per dish (Scr, *n =* 5; Scr + ET-1, *n =* 4; siFTO,*n =* 5; siFTO + ET-1, *n =* 5); ordinary two way-ANOVA with Tukey`s correction for multiple comparison was performed and significance levels were shown for each condition in comparison to the control. ***P < 0.001; ****p < 0.0001; **p < 0.005; *p < 0.05

Initially, we performed echocardiography for the FTOcKO and Cre control animals at three different age intervals (2,4 and 6 months), and as a consequence, FTOcKO mice in comparison to the Cre controls, showed significantly reduced ejection fraction (EF) and increased left ventricular interdimensional diameter (LVID) at the first 4 months of age which continued over time (Fig S1A,B). This indicates that FTOcKO mice have reduced cardiac efficiency and develops a dilatative phenotype as they age. To study cardiac adaptation, we performed TAC surgery in FTOcKO mice. 1-week post-TAC FTOcKO animals showed severe reduction in cardiac efficiency, with reduced ejection fraction (EF) and Fractional shortening (FS) compared to the Cre controls (Fig. 1A, B). Further, a higher degree of dilatation without change in intraventricular septum thickness (IVS) was observed in FTOcKO-TAC mice compared to Cre-TAC, whereas the control animals had no significant effect on LV dilatation after one week of TAC (Fig. 1C, D). And at the basal levels, there is a tendency of higher LVID;d in FTOcKO sham compared to Cre Sham (not significant) indicating that the lack of FTO undergoes a process of maladaptation and dilatation which is aggravated after TAC surgery. The LV posterior wall thickness (LVPW) is shown in Fig. 1 E. Although the ratio of LV/TL (LV weight to Tibia length) was increased in both FTOcKO TAC and control TAC groups (Fig. 1F), there was no increase in relative wall thickness (RWT) for FTOcKO TAC compared to the control TAC mice (Fig. 1G), indicating that FTOcKO mice develop rather dilated hypertrophy incontrast to the control mice that show concentric hypertrophy one-week post TAC. Further, myocardial hypertrophy were assessed by measuring the cross-sectional area of LV sections of control (sham, TAC) and FTOcKO (sham, TAC) mice using Wheat germ agglutinin (WGA) staining and in consistent with our echo data, FTOcKO TAC mice showed no induction in myocardial hypertrophy compared to the control TAC mice (Fig. 1H, I). This suggests that unlike control groups, FTOcKO animals surpasses the early-phase cardiac adaptive response and directly exhibit maladaptive cardiac remodeling with induced PO. Furthermore, the fibrosis staining performed using picro sirius red stain kit showed significantly increased fibrotic area (Fig. 1J, K) in the FTOcKO TAC groups compared to all other groups (cre Sham, Cre TAC, FtocKO Sham), indicating aggravated pathological remodelling under FTO knockdown in response to pressure overload. These findings characterize the FTOcKO TAC phenotype as maladaptive dilated remodeling without compensatory hypertrophy.

### FTO deficiency triggers cardiac atrophy and attenuates induced cardiac hypertrophy in human iPS-cardiomyocytes (hiPS-CMs)

As there is no compensatory hypertrophy over FTO ablation in vivo, we assessed if FTO has a direct effect on stress mediated hypertrophic response in vitro using human iPS-CMs*.* For this purpose, the hiPS-CMs were transfected either with Scr siRNAs or with siRNAs of FTO demethylase, and their hypertrophic response were investigated in the presence or absence of the peptide hormone, Endothelin-1 (ET-1), a well-known mediator of cardiac hypertrophy. As a result, the cell size measurement of the control hiPS-CM with ET-1 (Scr + ET-1) showed significantly increased hypertrophy,however, this hypertrophic effect was highly attenuated in the hiPS-CMs with FTO depletion (siFTO + ET-1) (Fig. 1L,M). Interestingly, the physiological growth of siFTO CMs is reduced in its cell-size compared to the control CM size, and this phenotype of limited cell-size at the basal level might be a vital factor for attenuated hypertrophic response upon ET-1 exposure (Fig. 1L,M). This shows that FTO has a direct effect on individual CM size, growth and stress induced compensatory hypertrophy.

### Deregulation of FTO-dependent m6A methylations deteriorate compensatory hypertrophy and provoke pathological remodeling

To further elucidate the necessity of FTO dependent m6A methylations and its regulatory mechanisms in stress induced cardiac hypertrophy, MeRIP sequencing was performed for the above analysed FTOcKO and Cre Control (Cre C) animals that underwent sham and TAC surgeries (1 week post-surgery), and the corresponding mice sample inputs were subjected to RNA seq to compare the changes of differential expression with differential methylation (Fig. S1C).

The RNA seq data showed global changes in the heart of FTOcKO (sham and TAC) and Cre C (sham and TAC) (Fig. S2 A-B); Predominantly, the FTO gene is downregulated in the heart of FTOcKO samples(sham and TAC) (Fig. S2 C -D), indicating the absolute knockout of FTO in the FTOcKO animals generated via Cre-loxp system. Further, we checked for the alterations in other m6A modifiers namely METTL3 (m6A methylase) and ALKBH5 (m6A demethylase) in the FTOcKO model. Our RNA-seq data and the western blot data (Fig. S3A-C), showed no significant changes in the key m6A modifiers indicating that FTO has no direct effect on the regulation of other m6A modifiers in the heart.

The global distribution of m6A peaks for each condition is showed in Fig. S4A.

**Fig. 2:**
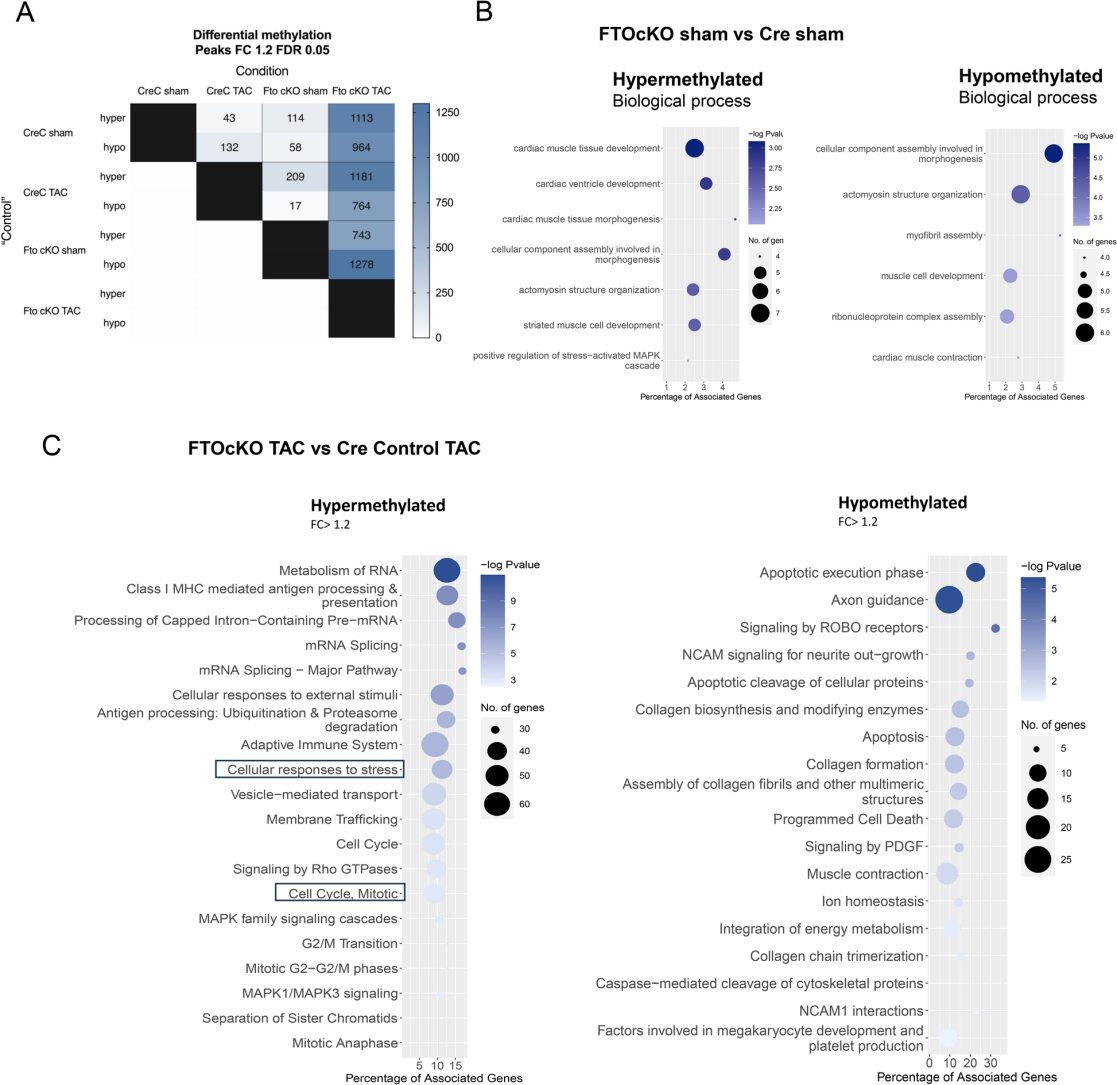
Differentially m6A methylated transcripts of FTO ablated animals. The MeRIP was performed for the heart tissues of FTOcKO and Cre control groups, hearts were isolated one week-post TAC surgery; (**A**) Detailed representation of differentially methylated transcript for each condition, CreC (sham& TAC) and FTOcKO (Sham &TAC), with FC1.5 FDR 0.05. (CreC: Cre Control group); (**B**) Gene Ontology enrichment of differentially methylated transcripts, FTOcKO Vs Cre control, with FC > 1.2 FDR 0.05: the biological process of differentially hypomethylated transcripts and differentially hypermethylated transcripts; (**C**) Biological pathways corresponding to differentially methylated transcripts in FTOcKO TAC group compared to CreC TAC mice groups

The transcripts that are differentially m6A methylated under the condition FTOcKO sham Vs CreCsham, are unique and regulate distinct signaling mechanisms incontrast to differentially expressed genes. The number of differentially m6A methylated transcripts (log2FC > 1.2; FDR 0.05) for each condition is listed in Fig. 2A. We detected around 114 differentially hypermethylated transcripts and 58 hypomethylated transcripts in FTOcKO sham compared to CreC sham. While hypermethylation can easily explained by the loss of FTO, hypomethylation might be due to secondary changes via effects on other m6A-modifying enzymes like (ALKBH5 or METTL3, METTL14). Comparable results have been found in other published studies [25–27]. Further, to check if the hypermethylation is caused only by the FTO loss, we validated in rescue experiments by performing dot blot assay for the global m6A changes in hiPS-CMs under Scr and siFTO conditions. Fig. S4B showed increased hypermethylation under FTO knockdown compared to the scr hiPS-CMs confirming the direct effect of FTO on the global m6A levels.

Interestingly, GO analysis showed that a higher percentage of differentially methylated transcripts in FTOcKO sham vs CreC Sham are linked to cardiac muscle tissue development, ventricle development, and cardiac muscle tissue morphogenesis (Myh7,Mybpc3,Tnnt2,Myl3) (Fig. 2B). Also the transcripts of mTORC1 signaling pathway (FNIP1, Akt1s1) and of the regulation of lysosomal biogenesis and autophagy (TFEB) were found to be differentially methylated.

Moreover, the differentially methylated transcripts exceeded the differentially expressed genes in FTOcKO TAC vs. CreC TAC (Fig. S4C), and higher percentage of differentially hypermethylated transcripts in FTOcKOTAC vs CreTAC are associated with cell cycle progression and mitosis, cell survival, apoptosis inhibition (namely, CFLAR and CDK1) (Fig. 2C). Further, in our methylation data, we detected increased hypomethylation in the collagen formation and organisation of extracellular and induction in apoptosis in both FTOcKO TAC vs. CreC TAC (Fig. 2C) and FTOcKO TAC vs. FTOcKO sham (Fig. S5A). This indicates that in the context of pressure overload (with TAC surgery), FTO loss increased collagen formation and exacerbation of apoptosis induction in the heart. Hence, the methylation deregulation-induced pathological turnover might be the basis for the impaired compensatory hypertrophy and maldaptive remodeling in FTO depleted hearts, eventually resulting in cardiac insufficiency and heart failure.

### FTO ablation in the heart has direct impact on cell survival, mTORC1 signaling and autophagy regulation

**Fig. 3:**
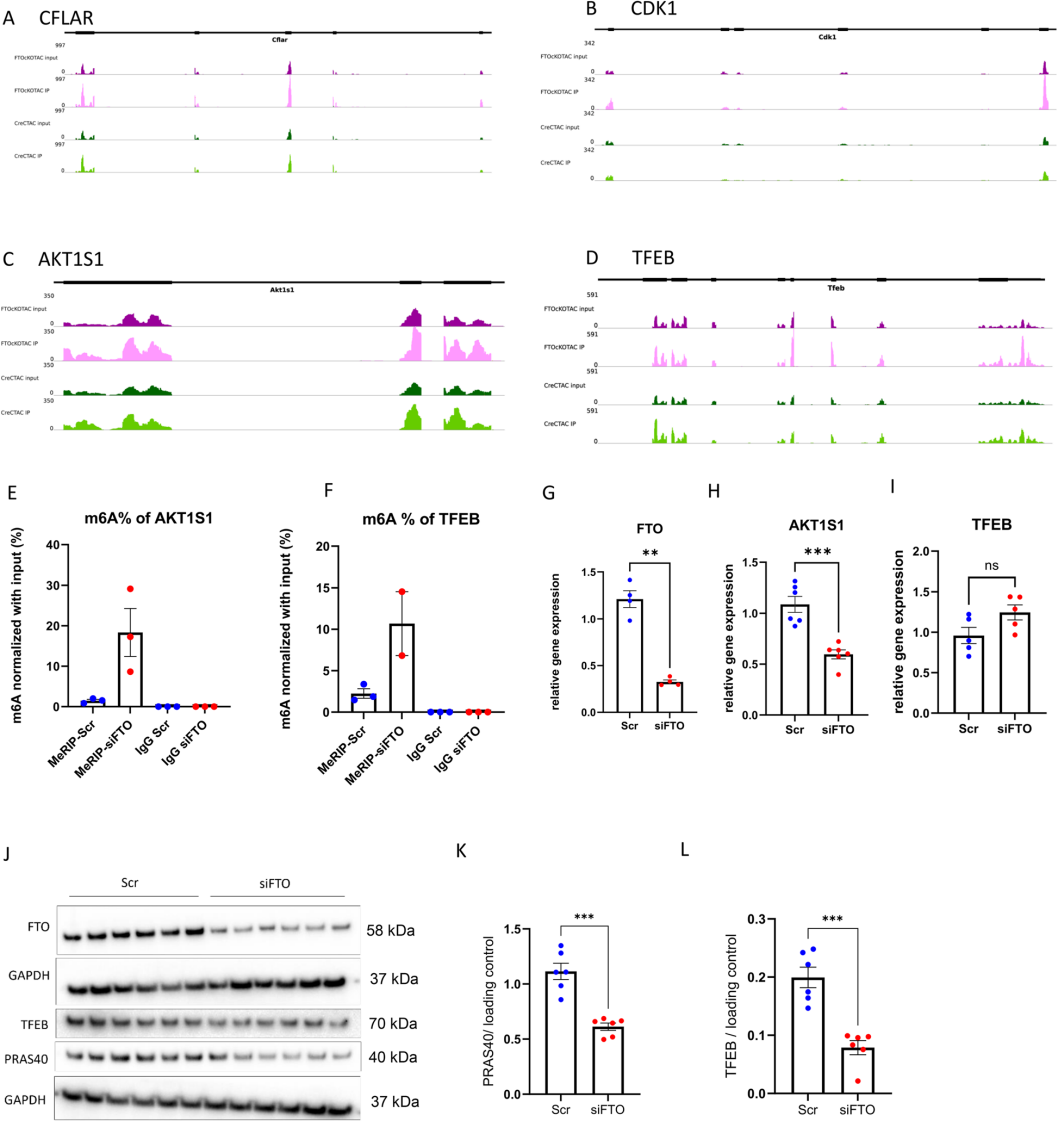
FTO ablation in the heart triggers hypermethylation in key transcripts of apoptosis inhibition, mTORC1-S6K1 signaling and autophagy: The transcription level m6A methylation changes are shown under conditions FTOcKO TAC vs CreC TAC for the transcripts (**A**) CFLAR, (**B**) CDK1, (**C**) AKT1S1 and (**D**) TFEB; MeRIP-qPCR was performed for the transcripts AKT1S1 and TFEB in scr and FTO-silenced hiPS-CMs, (**E**) shows the m6A enrichment of AKT1S1 and (**F**) shows the m6A enrichment of TFEB in MeRIP-siFTO compared to MeRIP-Scr, IgG Scr and IgG siFTO are the controls for m6A RNA immunoprecipitation; (**G**) mRNA expression data of FTO gene in Scr and siFTO hiPS-CMs; (**H**) relative gene expression of AKT1S1 in scr and siFTO hiPS-CMs; (**I**) relative gene expression of TFEB in scr and siFTO hiPS-CMs; (**J**) representative western blot image showing the signals of PRAS40 and TFEB proteins in Scr and siFTO-hiPS-CMs; (**K**) and (**L**) shows the pooled quantitative densitometry analysis for PRAS40 and TFEB respectively. The mean with SEM is presented for G-I and K-L, n represents no. of biological replicates; *n =* 4–6 for scr and *n =* 4–6 for siFTO hiPS-CMs; *p < 0.05; ** *p* < 0.005; *** < 0.001 all by two tailed unpaired t-test with Welch’s correction

Based on our MeRIP-seq data, deciphering the m6A methylome upon FTO knockdown is very complex as there are many transcripts that were hypermethylated, however, we aimed to target the transcripts that are involved in important signalling pathways. Notably, we detected hypermethylation in the key transcripts that are involved in 1. cell response to stress and apoptosis inhibition (CFLAR, CDK1), 2. mTORC1 signaling (AKT1S1) (Fig. S6A) and 3. autophagy regulation (TFEB). The transcript map shown in Fig. 3A-D highlights the hypermethylation of the transcripts especially near and within the exons under FTOcKO-TAC group compared to Cre TAC. Further, the hypermethylation of transcripts of mTORC1 pathway and autophagy were validated in hiPS-CMs model, Fig. 3E, F shows the increased m6A enrichment of AKT1S1 and TFEB in FTO knockdown cardiomyocytes compared to their respective controls. Furthermore, the mRNA expression data of AKT1S1 and TFEB under FTO knockdown condition (Fig. 3G, H, I) showed reduced AKT1S1 expression and no significant change in TFEB expression, however, their corresponding protein analysis data in Fig. 3J,K,L showed downregulation in both PRAS40 (encoded by AKT1S1) and TFEB total protein levels in FTO silenced cardiomyocytes. We next assessed whether the downregulation of total PRAS40 and TFEB proteins observed in the hiPS-CM model was also present in LV heart tissues from FTOcKO (Sham, TAC) and CreC (Sham, TAC) mice. Western blot analysis revealed a significant decrease in PRAS40 and TFEB protein levels in FTOcKO (Sham, TAC) animals compared with CreC Sham controls, consistent with our in vitro findings (Fig. S7A-C). These results indicate that FTO loss promotes hypermethylation at the post-transcriptional level, thereby impairing translational efficiency and reducing protein expression, independent of changes at the mRNA level.

For the AKT1S1 transcript, we compared expression between FTOcKO TAC and CreC TAC (Fig. 3C) as well as between FTOcKO TAC and FTOcKO Sham (Fig. S6B). Increased hypermethylation in FTOcKO TAC versus CreC TAC was comparable to that observed in FTOcKO Sham versus CreC Sham (Fig. S6A), indicating no additional methylation changes with TAC. In FTOcKO TAC versus FTOcKO Sham, a non-significant trend toward hypomethylation was observed (log2FC = –0.3; Fig. S6B). Altogether, our analysis indicates that FTO loss directly affects AKT1S1 and modulates the mTORC1 pathway, without additional impact under pressure overload.

### Cardiomyocyte-specific FTO depletion induces apoptosis

**Fig. 4:**
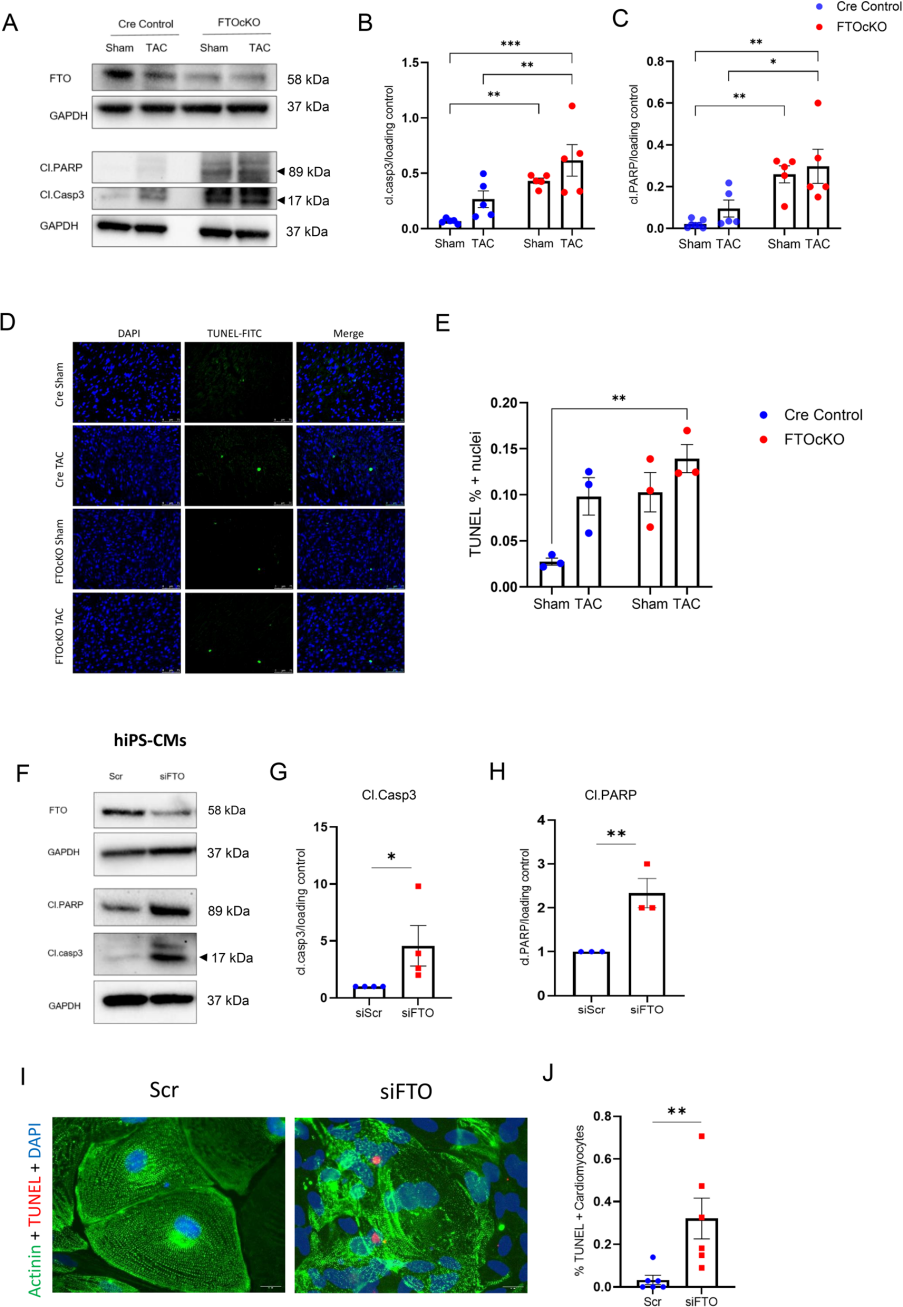
Loss of FTO demethylase induces cardiac apoptosis: (**A**) Representative western blots of Cre Control and FTOcKO heart samples with sham and TAC surgery. The apoptotic markers cleaved caspase 3 (cl.Casp3) and cleaved PARP (cl.PARP) were increased in FTOcKO Sham and TAC; (**B**) & (**C**) are pooled quantitative densitometry analysis for apoptotic markers cl.casp3 and cl.PARP respectively, from the heart samples of FTOcKO and Cre control groups (Sham &TAC); The mean with SEM is presented; n is no. of mice (CreC Sham, *n =* 4–5; CreC TAC, *n =* 5; FTOcKO Sham, *n =* 5; FTOcKO TAC, *n =* 5); (**D**) representative images of TUNEL staining of left ventricle (LV) sections from FTOcKO and Cre control animals (both Sham and TAC); (**E**) pooled TUNEL analysis, the percentage of TUNEL positive cells from the total number of cardiomyocytes were calculated; The mean with SEM is presented, mice condition (CreC Sham, *n =* 3; CreC TAC, *n =* 3; FTOcKO Sham, *n =* 3; FTOcKO TAC, *n =* 3); Statistical analysis (**B**, **C**, **E**) is by ordinary two-way ANOVA with Tukey’s correction for multiple comparisons; (**F**) Representative western blots of hiPS-CMs transfected with scr and FTO siRNAs; and the pooled densitometry analysis for cl.casp3 (**G**) and cl.PARP (**H**) were shown. **I** fluorescent double staining of alpha-actinin and TUNEL in hiPS-CMs with scr and siFTO silencing, and (**J**) represents the percentage of TUNEL positive cells (pooled data). The mean with SEM is presented. For G, H, J, biological replicate, *n =* 3–6 for each condition; For G & H, 1-sample t-test is performed, the results were normalized to the respective control conditions and ratios, in which a normal distribution of results cannot be proven, were analyzed. *p < 0.05; ** *p* < 0.005 all by unpaired t-test

As the MeRIP-seq analysis unfolded the defect in cell survival and apoptosis inhibition upon FTO loss, we analysed the effect of apoptosis in the LV heart tissues of FTOcKO (sham, TAC) and CreC (sham, TAC) mice samples. our western blot data showed a tendency of increase in apoptotic markers namely, cleaved caspase 3 (cl.casp3) and cleaved PARP (cl.PARP) in CreC TAC compared to CreC sham and statistically significant increase in FTOcKO (sham, TAC) compared to CreC sham (Fig. 4A-C). Further our TUNEL analyis of LV sections of FTOcKO and CreC animals (both sham and TAC) showed significantly increased apoptotic cells in FTOcKO TAC condition compared to CreC groups (Fig. 4D,E). The effect of apoptosis on FTOcKO sham and Cre TAC looked similar in our TUNEL data, indicating that FTO depletion and pressure overload has an impact on cell survival and further the combined effect of FTO loss and TAC surgery exacerbates apoptosis induction.

Furthermore, to identify whether the effect of FTO depletion on apoptosis is cardiomyocyte specific, we analysed the activity of cl.casp3 and cl.PARP in FTO silenced hiPS-CMs (Fig. 4F-H). Consequently, there was a significant upregulation in the apoptotic markers in siFTO-hiPS-CMs compared to the Scr-hiPS-CMs. Further, the TUNEL assay showed highly increased TUNEL positive cells in siFTO-hiPS-CM samples indicating the crucial effect of FTO loss on cardiomyocyte death (Fig. 4I,J).

### Hyperactivation of mTORC1 pathway in FTO-depleted cardiomyocytes

**Fig. 5:**
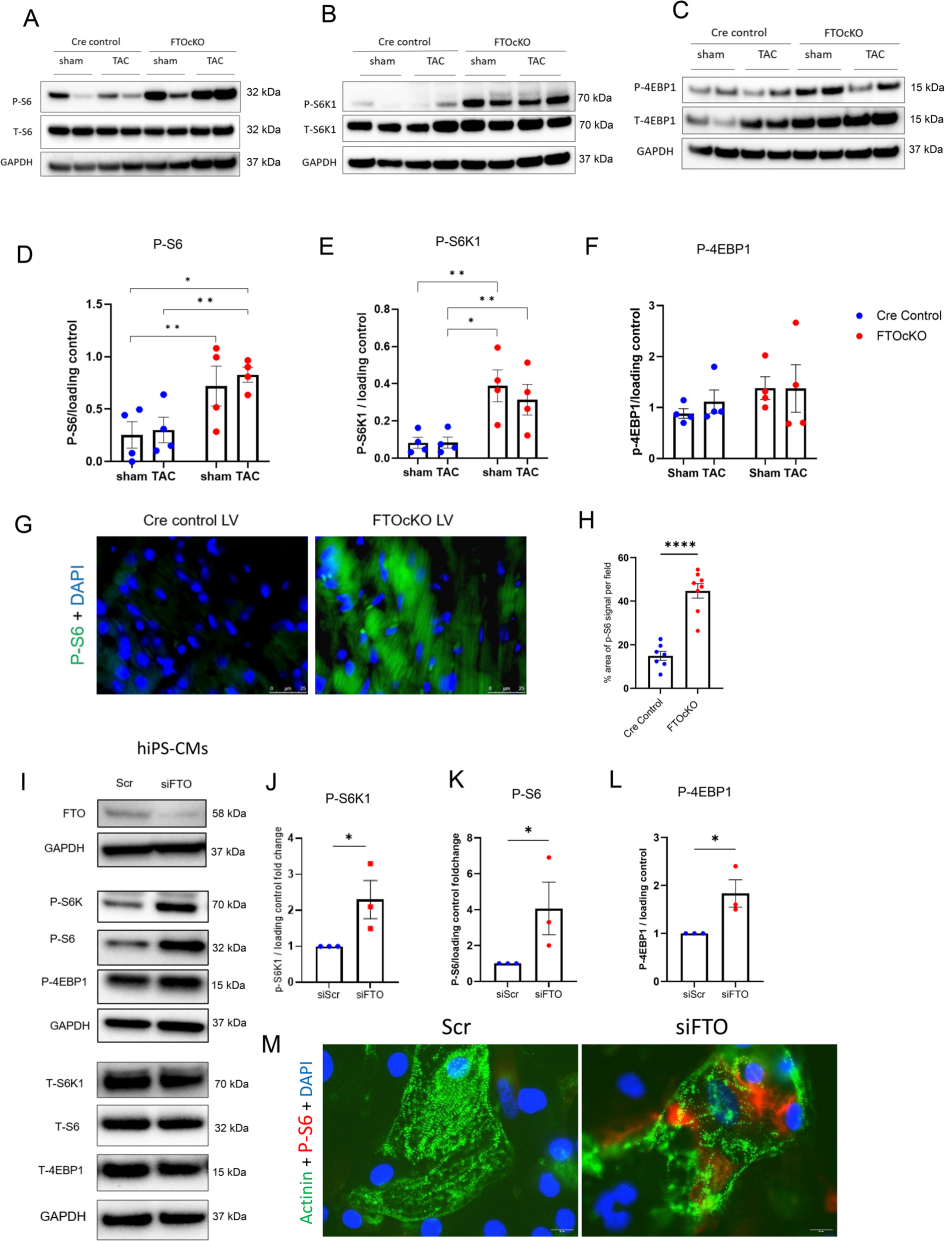
FTO depletion in cardiomyocytes significantly increased the mTORC1 signaling pathway: Representative western blots of Cre Control and FTOckO animals with Sham and TAC surgery. **A** The phosphorylation of S6 (ser235/236), (**B**) p70S6 kinase (thr389) and (**C**) 4EBP1 (Thr37/46) were increased in FTOcKO mice groups; pooled quantitative densitometry analysis of P-S6 (**D**), P-S6K1 (**E**), P-4EBP1 (**F**); The mean with SEM is presented; mice *n =* 4 for each condition; Statistical analysis (**D**, **E**, **F**) is by ordinary two-way ANOVA with Tukey’s correction for multiple comparisons; *p < 0.05, ** *p* < 0.005; (**G**) representative P-S6 (green) staining of FTOcKO and Cre control LV sections; (**H**) the percentage of p-S6 signal per field is analysed in CreC and FTOckO LV sections; The mean with SEM is presented; n represents each LV sections, *n =* 7–8 from 3 different mice; statistical analysis is by unpaired t-test with Welch’s correction, ****p < 0.0001; (**I**) Representative western blots of hiPS-CMs transfected with scr and siFTO; (**J**), (**K**) & (**L**) shows the pooled quantitative densitometry analysis of P-S6K1, P-S6 and P-4EBP1 respectively; (**M**) Fluorescent double staining of P-S6 (red) and alpha-actinin(sarcomeric) (green). The mean with SEM is presented; biological replicate, *n =* 3 for each condition; the results were normalized to the respective control conditions and ratios, in which a normal distribution of results cannot be proven, were analyzed *p < 0.05 all by unpaired t-test with Welch’s correction for J-L

After our meRIP-seq analysis of FTOcKO animals revealed hypermethylation in the transcript AKT1S1 and the downregulation of its protein PRAS40 which is a subunit of mTORC1 complex and a negative regulator of mTORC1 signaling, we then looked into whether mTORC1 pathway is dysregulated in the myocardium of FTO defective mice. The activity of mTORC1 is demonstrated by phosphorylation of its canonical substrates namely, phosphorylation of S6K1 at Thr 389 (pS6K1), phosphorylation of ribosomal protein S6 at ser235/236 (pS6) and phosphorylation of 4EBP1 at Thr37/46 (p-4EBP1). As a result, our western blot data showed significant increase in p-S6K1 and p-S6, and there was no statistical increase in p-4EBP1 in the myocardium of FTO-depleted mice (FTOcKO sham and TAC) compared to the control group (Fig. 5A-F). This suggests that mTORC1-S6K1 activity is substantially enhanced in FTOcKO animals (Fig. 5A, B, D, E). Moreover, ribosomal protein S6 (p-S6 ser235/236) activity was significantly elevated in FTOcKO LV sections compared to control sections (Fig. 5G, H), showing hyperactivation of the mTORC1-S6K1 signaling upon FTO depletion. Further to identify if the effect of FTO on mTORC1 regulation is cardiomyocyte specific, we investigated the activity of mTORC1 in FTO silenced hiPS-CMs (siFTO-hiPS-CMs). In consistent with our mice data, there was significant increase in the mTORC1 substrates (including p-4EBP1) in siFTO-iPS-CMs compared to the control iPS-CMs (Fig. 5I, J, K, L). Further, the immufluorescence data showed significant increase in p-S6 (ser235/236) signaling in siFTO-iPS-CMs in comparison with the control (Fig. 5M).

Further, we have also checked for the effect of mTORC2 in the hearts of FTOcKO animals (both sham and TAC) in comparison with their respective controls by directly analysing the activity of AKT (phosphorylation at ser473, pAKTs473) which is a canonical substrate of mTORC2. Our western blot data (Fig. S8A,B) showed a tendency of pAKT induction which is not significant under FTO knockdown, indicating no direct effect of FTO on mTORC2 signalling in the heart.

Altogether our data indicates the direct regulatory relationship of FTO on mTORC1-S6K1 pathway in the adult cardiomyocytes.

### FTO deficiency in hiPS-CMs causes impaired autophagic flux and inadequate autophagy.

**Fig. 6:**
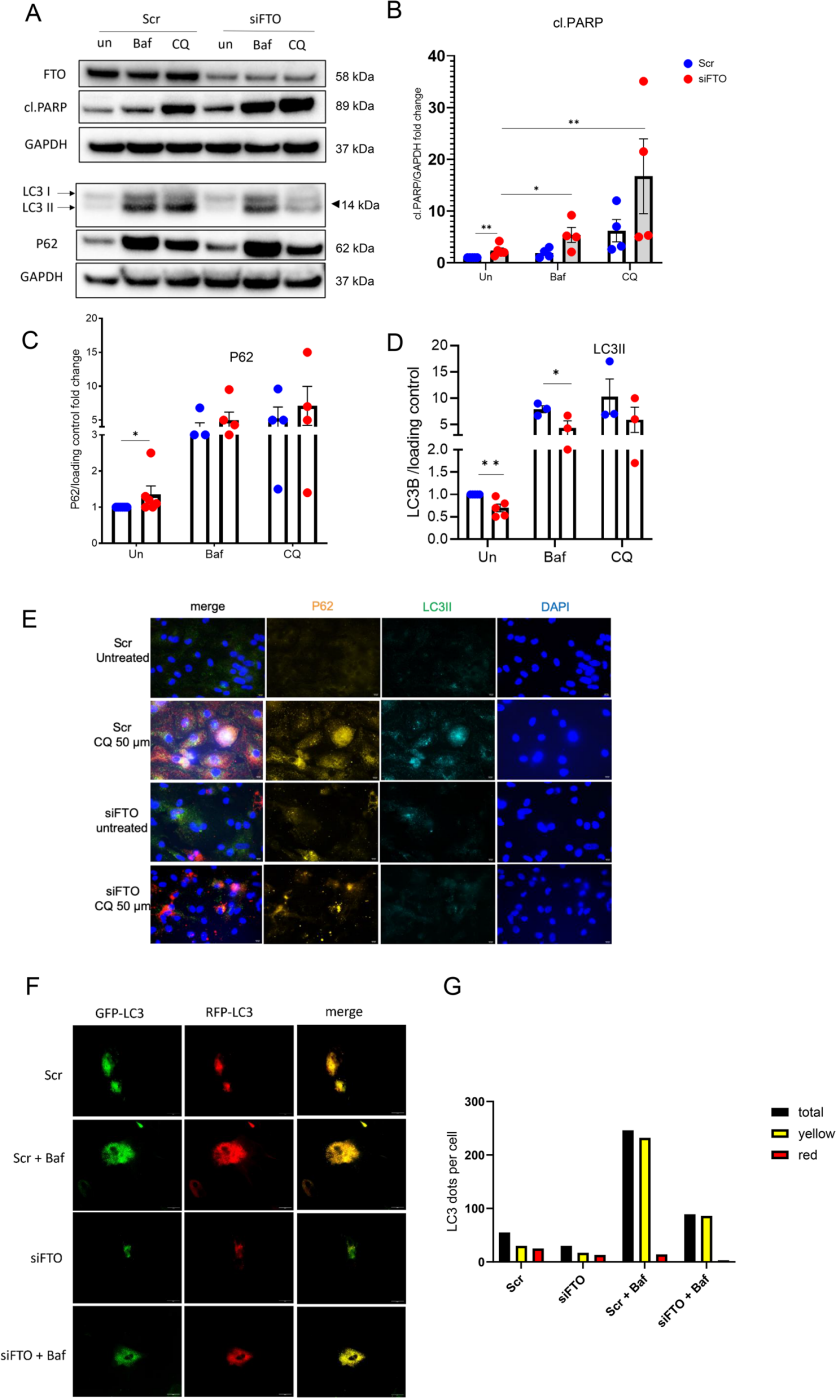
FTO depletion and inhibition of late autophagy exacerbates cardiac apoptosis and impairs autophagic flux: The hiPS-CMs were transfected with siRNAs of FTO and scr, and the late-autophagy (fusion of autophagosomes and lysosomes) were blocked by using the inhibitors, Bafilomycin A (Baf) and Chloroquine (CQ) with the final concentration of 50 nM and 50 uM, respectively for 16 h; (**A**) Representative western blots of the autophagic markers (LC3 II and P62) and apoptosis (cl.PARP); pooled quantitative densitometry analysis of cl.PARP (**B**), P62 (**C**) and LC3 II (**D**); (**E**) Representative fluorescent images of LC3 II and p62 co-localization (P62-red; LC3 II – green; DAPI-blue) for scr and siFTO iPS-CMs treated with or without 50uM of Chloroquine (CQ) for 16 h. The mean with SEM is presented. biological replicate, *n =* 3–5 for each condition; the results were normalized to the respective control conditions and ratios, in which a normal distribution of results cannot be proven, were analyzed ** *p* < 0.005; *p < 0.05 all by unpaired t-test; (**F**) represents the live imaging of tandem mRFP-GFP-LC3 assay, the hiPS-CMs were transduced with RP-LC3 for 24 h and further transfected with scr and FTO siRNAs before treatment with or without Baf for 12 h; (**G**) represents the quantified data of LC3 puncta per cell under each conditions (Scr, siFTO, Scr + Baf and siFTO + Baf), black indicates total LC3 puncta per cell; yellow represents no. of autophagosomes per cell and red represents no. of autolysosomes per cell; total of 15 to 30 cells were analysed per dish, in total 6 dishes were considered for each condition. The mean value of all the cells for each condition were plotted in graph

As our MeRIP data showed hypermethylation in TFEB transcript under FTO knockdown and downregulation of TFEB at the protein levels, we assume that the dysfunctional TFEB and hyperactive mTORC1 affects autophagy in the FTO silenced cardiomyocytes. Hence, to comprehend how FTO deletion impacts autophagic flux in cardiomyocytes, we suppressed late-stage autophagy in hiPS-CMs transfected with or without siFTO by using the well-known inhibitors bafilomycinA1 (Baf) and chloroquine (CQ). As anticipated, Baf and CQ's suppression of the autophagic flux led to the induction of apoptosis, which was further exacerbated by FTO silencing (Fig. 6A, B). Further, the autophagic flux was measured by the protein levels of the autophagic markers ubiquitin-binding scaffold protein p62 (also known as SQSTM1) and microtubule-associated protein 1A/1B-light chain 3 (LC3-II). As a result, p62 significantly increased and LC3II significantly decreased in FTO silenced hiPS-CMs at the basal level compared to the scr-hiPS-CMs, indicating defective autophagy upon FTO deficiency in cardiomyocytes (Fig. 6A, C, D). Moreover, hiPS-CMs treated with Baf and CQ accumulated more LC3II and p62 than control, indicating that autophagy was being suppressed (Fig. 6A, C, D). However, this effect was different in siFTO-hiPS-CMs with Baf and CQ compared to its respective control (Scr-hiPS-CMs with Baf and CQ) as there was a decrease in LC3II (not significant for CQ condition) without comparative changes in p62 accumulation, which suggested a disruption in steady-state autophagosome formation (Fig. 6A, C, D). To further determine the autophagic flux, the colocalization of p62 and LC3 puncta was assessed in FTO knockout hiPS-CMs treated with or without CQ. We detected increased p62 accumulation in FTO knocked out cardiomyocytes compared to the Scr (Fig. 6E). Also, there was increased p62 and LC3II colocalization in scr-hiPS-CMs with CQ indicating increased autophagic flux and autophagosome induction, however, this effect was attenuated in FTO silenced CMs with CQ (siFTO-iPS-CMs + CQ) which showed less induction in LC3II and reduced colocalization of p62 and LC3II (Fig. 6E).

Moreover, we performed the tandem mRFP-GFP-LC3 assay in hiPS-CMs with or without FTO knockdown to analyse the direct effect of FTO on autophagic flux. The representative figures of the RFP-GFP-LC3 puncta under each condition (Scr, Scr + BAf, siFTO, siFTO + Baf) are shown in Fig. 6F and the quantified data Fig. 6G shows the average total number of LC3 puncta per cell in black, number of autophagosomes per cell in yellow and number of autolysosomes per cell in red. Our data shows that the total number of autophagosomes (shown as yellow dots in 6G) is reduced upon FTO knockdown both at the basal level and with the bafilomycin treatment compared to the Scr, indicating defect in the formation of autophagosomes and subsequent autophagy impairment. The above assay corroborates the western blot and LC3-P62 immunofluorescence data. This suggests that autophagic flux is impaired and the autophagosome induction is defective in FTO depleted cardiomyocytes. Therefore, our data show that FTO demethylase plays a crucial part in controlling autophagy in cardiomyocytes.

### Pharmacological inhibition of mTORC1-S6K1 pathway is sufficient to restore cardiomyocyte survival and ameliorate cardiac atrophy

**Fig. 7:**
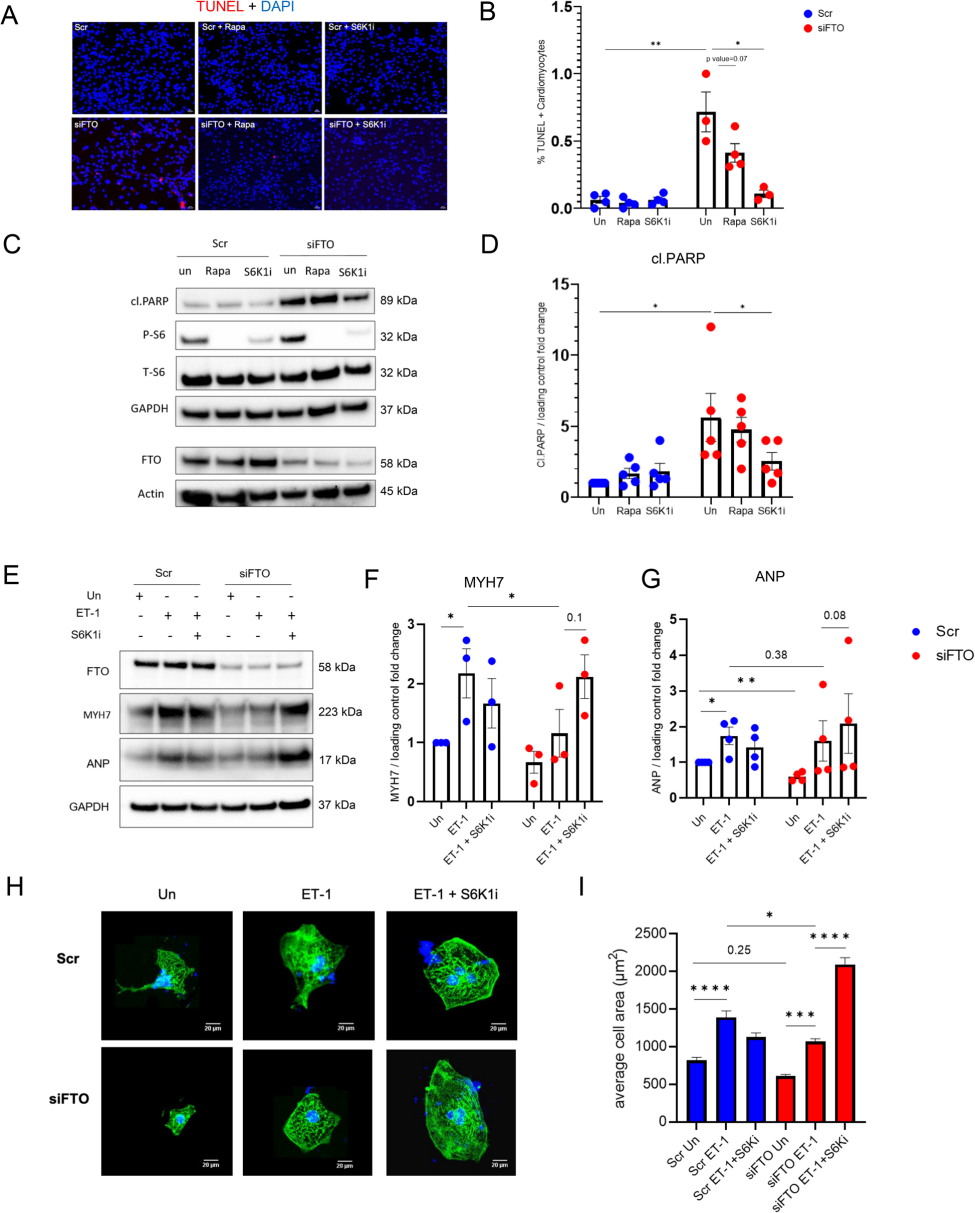
FTO ablation induces apoptosis and atrophy via mTORC1-S6K1 pathway: The hiPS-CMs were transfected with siRNAs of FTO and scr for 24 h, further treated with mTORC1 inhibitors Rapamycin (Rapa) and PF-4709671(S6Ki) with 50 nM and 5 µM final concentration respectively, for another 24 h: (**A**) Representative images of TUNEL staining for each condition (Un – Untreated); (**B**) pooled TUNEL analysis, the percentage of TUNEL positive cells from the total number of cardiomyocytes were calculated for each condition, biological replicate, *n =* 3–4 for each condition; (**C**) Representative western blots of FTO silenced hiPS-CMs with or without mTORC1 inhibitors, the activity of mTORC1-S6K1 is showed by P-S6 signals; cl.PARP signal represents cardiac apoptosis; (**D**) pooled quantitative densitometry analysis of cl.PARP (marker of apoptosis); biological replicate, *n =* 5 for each condition; *p < 0.05 and **p < 0.01 all by student’s t-test; (**E**-**I**) Study of the effect of hypertrophic response by Endothelin-1(ET-1) in siFTO-hiPS-CMs and scr hiPS-CMs with or without PF-4709671 (S6K1i); 24 h after transfection, the CMs were incubated with or without 10 nM of ET-1 and 5 µM of S6K1i for 24 h respectively: (**E**) Representative western blots of cardiomyocyte hypertrophic markers, MYH7 and ANP; (**F**) and (**G**) shows the pooled quantitative densitometry analysis of MYH7 and ANP respectively, The mean with SEM is presented. biological replicate, *n =* 3–4 for each condition; *p < 0.05 and **p < 0.01 all by student’s t-test; (**H**) Immunofluorescence data showing the effect of ET—1 and S6K1i in siFTO-CMs and scr-CMs (alpha-actinin in green and DAPI in blue); (**I**) Measurement of average cell area in µm.^2^ quantified using Image J; The mean with SEM is presented. n represents single cell (Scr Un, *n =* 167; Scr ET-1, *n =* 116; Scr ET1 + S6K1i, *n =* 92; siFTO Un, *n =* 103; siFTO ET-1, *n =* 94; siFTO ET-1 + S6K1i, *n =* 166) statistical analysis is performed using one-way ANOVA column analysis *p < 0.05; *** *p* < 0.001; **** *p* < 0.0001

We further analysed the FTO-mTORC1 crosstalk in the regulation of apoptosis and cardiac hypertrophy. Firstly, to identify if FTO regulate apoptosis through mTORC1 pathway, we blocked mTORC1-S6K1 signaling by the use of selective inhibitors against mTORC1 (Rapamycin) and S6K1 (PF-4708671 a.k.a. S6K1i) in FTO silenced hiPS-CMs and checked the effect of apoptosis. As a result, rapamycin and S6K1i completely suppressed mTORC1 activity as shown by p-S6 (Fig. 7 C) and interestingly, reduced the induction of apoptosis as shown by apoptotic marker cl.PARP in Fig. 7C-D and TUNEL assay (Fig. 7A, B). Moreover, we observed highly reduced apoptosis induction in siFTO-hiPS-CMs treated with S6K1i compared to rapamycin(Rapa), demonstrating that mTORC1-S6K1 signaling is involved in the onset of apoptosis.

Secondly, to identify if the cardiac atrophy caused upon FTO loss could be reversed by mTORC1-S6K1 inhibition, we checked for the hypertrophic markers namely, MYH7 (β-myosin heavy chain) and ANP (atrial natriuretic peptide) in FTO silenced hiPS-CMs with or without mTORC1-S6K1 inhibition. Consequently, the significant decrease in ANP and MYH7 protein levels that followed FTO ablation was significantly restored by S6K1i treatment and not by Rapamycin (Fig. S9 A-C).

Further, we investigated whether the mTORC1-S6K1 inhibitor S6K1i could restore the attenuated ET-1-induced-hypertrophic response in FTO knockdown hiPS-CMs. As a result, our western blot data showed that S6K1i could significantly increase the hypertrophic marker, MYH7 protein levels in siFTO-ET-1 CMs (Fig. 7E-G); and Immunofluorescence data showed that S6K1i could significantly rescue the ET-1-induced hypertrophic response in FTO silenced CMs (Fig. 7H, I). This implies that FTO-mTORC1-S6K1 crosstalk is necessary for both cardiac survival and cardiac hypertrophy.

Overall, our data suggests that loss of FTO induces apoptosis and shrinks cardiomyocyte via the unusual hyperactivation of mTORC1-S6K1 signaling pathway in hiPS-CMs, and pharmacological inhibition of mTORC1-S6K1 pathway is sufficient to reverse the pathological remodeling of FTO loss and m6A deregulation.

## Discussion

In this study, we show that cardiac early adaptation critically depends on a functional m6A RNA methylation system. Loss of FTO leads to rapid progression into severe heart failure, shifting cardiomyocyte remodeling from hypertrophy toward atrophy and apoptosis, driven by mTORC1–S6K1 hyperactivation and impaired autophagy, while pharmacological inhibition of mTORC1–S6K1 rescues the pathology. Although FTO-mediated m6A modification has been linked to cardiac remodeling and heart failure in previous studies, the novelty of our work lies in dissecting the *early phase* of pressure overload–induced remodeling. By applying a one-week TAC model, we capture the critical transition from adaptive to maladaptive remodeling, a stage largely overlooked in earlier work that focused on chronic or established heart failure.

Further, we employed hiPSC-CMs rather than rodent primary cardiomyocytes to enable mechanistic investigations directly in a human system with high translational relevance. hiPSC-CMs provide a robust and reproducible source of highly pure (> 95%) cardiomyocytes with validated electrophysiological properties, and their essentially unlimited availability allows diverse experimental applications. In our study, they served as a suitable platform to dissect FTO-dependent signaling pathways in a human context, complementing the in vivo findings. While hiPSC-CMs are immature and cannot reproduce mechanical load, they are widely used and accepted to complement animal studies and dissect signaling pathways [28, 29].

### m6A methylations are crucial for adaptive cardiac hypertrophy

The response to increased afterload at first involves hyperacute adaptation of calcium cycling by CaMKII-activation, followed by compensatory hypertrophy in the first days [30, 31]. it is demonstrated that this early compensatory phase has favourable adjustments in Ca2 + sensitivity as well as preserved contractile output which is deteriorated at the later stages [1, 32]. Indeed, our echo data showed that our control mice developed concentric hypertrophy with preserved cardiac output one-week after TAC surgery. Nevertheless, in FTOcKO mice, this early compensatory hypertrophy is absolutely lost, and they instead have a dilatative phenotype with decreased cardiac output. This shows that disturbance of m6A-RNA-methlyation by FTO-knockout shifts from the adaptive to a maladaptive response after pressure overload. At one-week post-surgery, compensated hypertrophy is observed, followed by a progressive development of heart failure at extended TAC durations, specifically at eight weeks after TAC surgery. Our previous study on cardiac hypertrophy (1-week post TAC) showed increased hypomethylation in the pathways of cellular response to stress, and metabolic processes, further, in the heart failure model (8-week post TAC), we detected increased methylation changes in metabolic processes and mitochondrial functions that triggers increased stress in the heart [14]. However, as demonstrated in this study, in our FTOcKO model, we found hypermethylation in the development of cardiac muscle and impairment in the cellular response to stress one week after TAC, indicating failure in the early adaptive process. Additionally, the hearts of FTOcKO animals experienced a higher reduction in EF (< 10%) and a quicker decompensation within four weeks after TAC, indicating a faster progression of heart failure in response to pressure overload [14].

The influence of FTO on adaptive hypertrophy was also demonstrated here in iPS-cardiomyocytes. In the long run, lack of adaptive signaling exacerbates with pressure overload and induces heart failure in FTOcKO mice with increased collagen formation. Most heart failure patients have – even when ejection fraction is reduced – episodes of increased afterload. FTO downregulation in these patients drives maladaptive signaling by these episodes eventually explaining the decompensation-triggered progression of heart failure [33].

In the context of m6A RNA methylation, heart failure is characterized by marked increase in global m6A methylation and steady downregulation in FTO expression in both rodents and human failing hearts [14, 17, 34–36]. Further, it is also shown that METTL3 is indespensible for cardiac homeostasis and cardiac hypertrophy, indicating the importance of both m6A methylase (e.g. METTL3) and demethylase (FTO) in cardiac function.

In this current study, at the basal level, we demonstrated that FTO-deficient mouse hearts had decreased cardiac performance and developed a dilatative phenotype by the age of 4 months which worsened at the time of 6 months. This finding contrasts with that of Dorn et al., who showed that METTL3-cKO animals exhibit cardiac anomalies at the beginning of the eight-month period [5]. Although METTL3 is crucial for cardiac homeostasis and hypertrophy, its absence might be compensated by other m6A modifiers in order to balance its m6A regulatory effects. However, this compensatory effect is likely to be limited in FTOcKO animals which results in rapid downfall in cardiac function and increased cardiac dilation. Hence, the dilatative phenotype of FTOcKO mice exacerbates with pressure overload, ultimately resulting in rapid heart failure within 4–6 weeks post-TAC surgery. In vitro, we showed that FTO depletion in cardiomyocytes reduced the cardiomyocyte geometry and further with ET-1 treatment showed attenuated hypertrophy. The similar effect of reduced hypertrophy when exposed to hypertrophic stimuli was demonstrated using m6A methylase METTL3 knockdown in primary cardiomyocytes [5]. However, unlike FTO knockdown, METTL3 silencing had no influence on basal cardiomyocyte growth. Furthermore, through our MeRIP-seq analysis we identified that the transcripts of cardiac muscle tissue development, ventricle development, and cardiac muscle tissue morphogenesis are differentially m6A methylated with higher percentage in FTO knockout rodent hearts, indicating the tight regulation of FTO demethylase on the mRNA transcripts of cardiac hypertrophy and remodeling.

### Diminished FTO triggers deleterious signaling mechanisms.

Furthermore, FTO deficiency not only led to m6A imbalance in the mRNA transcripts causing both hyper and hypo methylations, but also activated deleterious signaling pathways in the rodents hearts and human iPS-CMs as a consequence.

We know that deciphering the m6A methylome is complex and it involves quick changes in the transcripts under stress and influences the translational efficiency with or without correlation at the mRNA levels. However, based on our methylation data and mechanistical investigations, we hypothesise that the increased stress and apoptosis induction in the heart of FTOcKO TAC animals compared to control groups are primarily caused by 1. hypermethylation in the transcripts of apoptosis regulation (CDK1, CFLAR), 2. hypermethylation in the transcript involved in mTORC1 signaling (AKT1S1), which causes mTORC1 hyperactivation, and 3. hypermethylation of the transcript involved in autophagy regulation (TFEB), which causes autophagy impairment.

In vitro, FTO loss induced apoptosis in cardiomyocytes indicating its cardioprotective effect and control over pathological remodeling. This is consistent with recent publications that showed FTO to have anti-apoptotic and anti-inflammatory effects on cardiomyocytes exposed to hypoxia/reoxygenation (H/R) [37, 38].

Further, we discovered improper hyperactivation in mTORC1-S6K1 pathway in FTO deficient cardiomyocytes. There were more studies on the effect of mTORC1 on cardiac hypertrophy [21, 22, 39]. Although it is known that mTORC1 signaling mechanism is essential for cardiovascular development during embryogenesis and the postnatal stage [22, 23, 40, 41], many pieces of evidence show that partial inhibition of mTORC1 improves cardiac function and reverses the maladaptive cardiac remodeling by reducing hypertrophy and fibrosis in ageing and in pressure overload model [22, 42–45]. Moreover, mTORC1 is primarily dysregulated and maladpative in a number of pathological conditions such as cancer, neurological disorders, obesity, and T2D [46]. Further, constitutive activation of S6K1 increase oxidative stress, mitochondrial dysfunction and cause early senescene in endothelial cells [47]. Correspondingly, there are evidences on inappropriate activation of mTORC1 and reduced autophagy in the mouse models of cardiomyopathy, and further, mTORC1 inhibition by rapamycin rescued the cardiac function in these animals [48, 49].

In relation to this, our meRIP data from the heart of FTOcKO mice indicated hypermethylation in the transcripts of AKT1S1 (encodes PRAS40) which is a binding partner and regulator of mTORC1 activity, and hypomethylation in the transcripts of FNIP1 which is the regulator of S6K1 phosphorylation [50, 51]. We identified that FTO knockdown in cardiomyocytes has impact on AKT1S1 both at the mRNA and protein levels. The mRNA expression of AKT1S1 is significantly reduced and further the hypermethylation of AKT1S1 reduced the translational efficiency reflecting in the downregulation of PRAS40 total protein levels in FTO depleted cardiomyocytes. Therefore, reduction in PRAS40 total protein might be the major contributing factor for mTORC1-S6K1 hyperactivation in the FTO silenced cardiomyocytes. Altogether, our study discloses the possible fact that m6A imbalance under FTO loss, subsequently caused dysfunctional mTORC1-S6K1 pathway, resulting in pathological remodeling in the heart.

The role of m6A alterations in controlling the lysosomal degradatory pathway autophagy has been well described in different cell types. Despite disagreements regarding the relationship between FTO and the autophagy pathway in cancer cells [52–54], two significant investigations showed the beneficial regulatory effect of FTO demethylase on autophagy [55, 56].

Consequently, we discovered the FTO demethylase's potent regulatory influence on the induction of autophagy in cardiomyocytes. The production of autophagosomes was reduced and the auophagic flux was inadequate due to FTO deficiency in cardiomyocytes which is in correlation with Jin et al., where they showed using HEK293T cells and HeLa cells that FTO loss reduced autophagy induction by reducing ULK-1 protein levels and LC3 puncta respectively [55, 57]. Furthermore, we detected differential methylation in TFEB transcript in the heart of FTOcKO mice. The transcription factor,TFEB is known to regulate the expression of genes involved in autophagy initiation, autophagosome formation and lysosomal biogenesis [58]. In our study, TFEB is hypermethylated upon FTO loss influencing the translational efficiency resulting in reduced total protein levels of TFEB without significant changes at the mRNA levels. The defective autophagy in FTO knockdown cardiomyocytes may therefore be caused by a combination of mechanisms, including hypermethylation of TFEB transcripts and increased mTORC1-S6K1 signaling via AKT1S1 hypermethylation.

### Inhibition of mTORC1-S6K1 is sufficient to reverse pathological remodeling

Furthermore, in FTO-depleted human cardiomyocytes, the effects of cardiac atrophy and apoptosis were effectively counteracted by PF-4708671, a selective S6K1 inhibitor. It should be noted that overexpression of FTO in the heart failing models is undoubtedly a potential therapeutic target as shown by Mathiyalagan et al.; However, the process of heart-specific gene therapy development (eg. AAV mediated gene therapy, RNA-targeted therapy) to increase FTO protein expression is firstly, time consuming and has its own limitations. In this line, our findings suggest a novel downstream target, the mTORC1-S6K1, in the course of pathological remodeling. Clinical research investigations have already developed promising and pharmacologically available p70S6K1 small molecule inhibitors namely, LY2584702 [59], DG2 [60] and AT7867 [61] which have impressive pre-clinical data for efficacy and safety for the treatment of cancer [62]. Therefore small molecule inhibitors and FDA approved mTOR inhibitors targeting mTORC1-S6K1 might be an interesting target for further heart failure therapy.

## Limitations

One limitation of our study is that sample sizes were based on prior experimental experience rather than formal power analysis. As a result, some variability exists between groups, particularly between Sham and TAC animals. While the sample sizes were sufficient to detect robust differences in most measured parameters, this variability may have affected the statistical power for certain comparisons and should be considered when interpreting the results.

## Conclusions

**Fig. 8:**
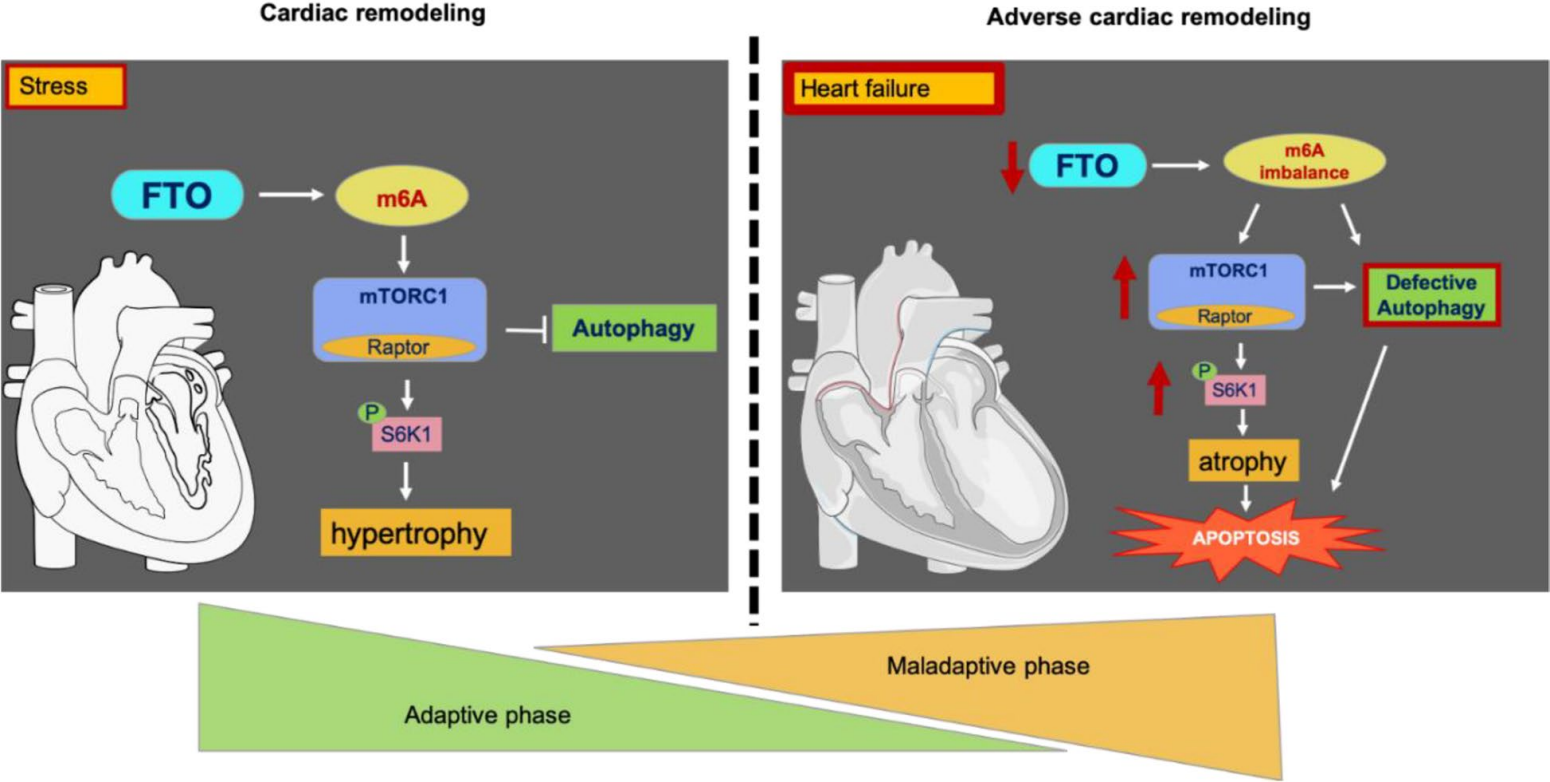
Proposed model of regulatory effects of FTO dependent m6A methylation in cardiac remodeling: Under acute stress, FTO dependent m6A methylations cordially regulate mTORC1-S6K1 pathway to conduct cardiac hypertrophy which is the early stage of adaptive cardiac remodeling. However, under chronic stress conditions, FTO is diminished and m6A network is imbalanced; mechanistically, FTO deficiency increases mTORC1-S6K1 hyperactivation and defective autophagy subsequently inducing cardiac atrophy and apoptosis, eventually heart failure

In conclusion, our research sheds light on the significance of FTO-dependent m6A methylations in coordinating the signaling processes that lead to early compensatory hypertrophy (Fig. 8). Also, we uncovered the detrimental pathways of pathological remodeling in relation to the imbalance in the FTO-m6A pathway and its role in heart failure (Fig. 8). FTO demethylase and its underlying functional mechanisms may thereby pave the way for novel therapeutic approaches to the early phase therapy of cardiac remodeling and heart failure.

## Supplementary Information

Supplementary Material 1

Supplementary Material 2

## Data Availability

No datasets were generated or analysed during the current study.

## References

[1] Pitoulis FG, Terracciano CM. Heart plasticity in response to pressure- and volume-overload: a review of findings in compensated and decompensated phenotypes. Front Physiol. 2020;11:92. 32116796 10.3389/fphys.2020.00092 PMC7031419

[2] Shimizu I, Minamino T. Physiological and pathological cardiac hypertrophy. J Mol Cell Cardiol. 2016;97:245–62. 27262674 10.1016/j.yjmcc.2016.06.001

[3] Oldfield CJ, Duhamel TA, Dhalla NS. Mechanisms for the transition from physiological to pathological cardiac hypertrophy. Can J Physiol Pharmacol. 2020;98(2):74–84. 31815523 10.1139/cjpp-2019-0566

[4] Nakamura M, Sadoshima J. Mechanisms of physiological and pathological cardiac hypertrophy. Nat Rev Cardiol. 2018;15(7):387–407. 29674714 10.1038/s41569-018-0007-y

[5] Dorn LE, Lasman L, Chen J, Xu X, Hund TJ, Medvedovic M, et al. The N(6)-methyladenosine mRNA methylase METTL3 controls cardiac homeostasis and hypertrophy. Circulation. 2019;139(4):533–45. 30586742 10.1161/CIRCULATIONAHA.118.036146 PMC6340720

[6] Liu C, Gu L, Deng W, Meng Q, Li N, Dai G, et al. N6-methyladenosine RNA methylation in cardiovascular diseases. Front Cardiovasc Med. 2022;9:887838. 35571209 10.3389/fcvm.2022.887838 PMC9098837

[7] Zhang L, Hou C, Chen C, Guo Y, Yuan W, Yin D, et al. The role of N(6)-methyladenosine (m(6)A) modification in the regulation of circRNAs. Mol Cancer. 2020;19(1):105. 32522202 10.1186/s12943-020-01224-3 PMC7285594

[8] Zhao K, Yang CX, Li P, Sun W, Kong XQ. Epigenetic role of N6-methyladenosine (m6A) RNA methylation in the cardiovascular system. J Zhejiang Univ Sci B. 2020;21(7):509–23. 32633106 10.1631/jzus.B1900680 PMC7383322

[9] Dominissini D, Moshitch-Moshkovitz S, Schwartz S, Salmon-Divon M, Ungar L, Osenberg S, et al. Topology of the human and mouse m6A RNA methylomes revealed by m6A-seq. Nature. 2012;485(7397):201–6. 22575960 10.1038/nature11112

[10] Zhang C, Fu J, Zhou Y. A review in research progress concerning m6A methylation and immunoregulation. Front Immunol. 2019;10:922. 31080453 10.3389/fimmu.2019.00922 PMC6497756

[11] He PC, He C. M(6) a RNA methylation: from mechanisms to therapeutic potential. EMBO J. 2021;40(3):e105977. 33470439 10.15252/embj.2020105977 PMC7849164

[12] Dai XY, Shi L, Li Z, Yang HY, Wei JF, Ding Q. Main N6-methyladenosine readers: YTH family proteins in cancers. Front Oncol. 2021;11:635329. 33928028 10.3389/fonc.2021.635329 PMC8076607

[13] Zhou J, Wan J, Gao X, Zhang X, Jaffrey SR, Qian SB. Dynamic m(6)A mRNA methylation directs translational control of heat shock response. Nature. 2015;526(7574):591–4. 26458103 10.1038/nature15377 PMC4851248

[14] Berulava T, Buchholz E, Elerdashvili V, Pena T, Islam MR, Lbik D, et al. Changes in m6A RNA methylation contribute to heart failure progression by modulating translation. Eur J Heart Fail. 2020;22(1):54–66. 31849158 10.1002/ejhf.1672

[15] Fernandez Rodriguez G, Cesaro B, Fatica A. Multiple roles of m6A RNA modification in translational regulation in cancer. Int J Mol Sci. 2022;23(16):8971. 10.3390/ijms23168971 PMC9408962 36012237

[16] Kmietczyk V, Riechert E, Kalinski L, Boileau E, Malovrh E, Malone B, et al. m(6)A-mRNA methylation regulates cardiac gene expression and cellular growth. Life Sci Alliance. 2019;2(2):e201800233. 10.26508/lsa.201800233 PMC6458851 30967445

[17] Mathiyalagan P, Adamiak M, Mayourian J, Sassi Y, Liang Y, Agarwal N, et al. FTO-dependent n(6)-methyladenosine regulates cardiac function during remodeling and repair. Circulation. 2019;139(4):518–32. 29997116 10.1161/CIRCULATIONAHA.118.033794 PMC6400591

[18] Castro-Hernandez R, Berulava T, Metelova M, Epple R, Pena Centeno T, Richter J, et al. Conserved reduction of m(6)A RNA modifications during aging and neurodegeneration is linked to changes in synaptic transcripts. Proc Natl Acad Sci U S A. 2023;120(9):e2204933120. 36812208 10.1073/pnas.2204933120 PMC9992849

[19] Fan S, Hu Y. Role of m6A methylation in the occurrence and development of heart failure. Front Cardiovasc Med. 2022;9:892113. 35811741 10.3389/fcvm.2022.892113 PMC9263194

[20] Xu ZY, Jing X, Xiong XD. Emerging role and mechanism of the FTO gene in cardiovascular diseases. Biomolecules. 2023;13(5):850. 10.3390/biom13050850 PMC10216201 37238719

[21] Sciarretta S, Forte M, Frati G, Sadoshima J. New insights into the role of mTOR signaling in the cardiovascular system. Circ Res. 2018;122(3):489–505. 29420210 10.1161/CIRCRESAHA.117.311147 PMC6398933

[22] Sciarretta S, Volpe M, Sadoshima J. Mammalian target of rapamycin signaling in cardiac physiology and disease. Circ Res. 2014;114(3):549–64. 24481845 10.1161/CIRCRESAHA.114.302022 PMC3995130

[23] Shende P, Plaisance I, Morandi C, Pellieux C, Berthonneche C, Zorzato F, et al. Cardiac raptor ablation impairs adaptive hypertrophy, alters metabolic gene expression, and causes heart failure in mice. Circulation. 2011;123(10):1073–82. 21357822 10.1161/CIRCULATIONAHA.110.977066

[24] Kleinsorge M, Cyganek L. Subtype-directed differentiation of human iPSCs into atrial and ventricular cardiomyocytes. STAR Protoc. 2020;1(1):100026. 33111079 10.1016/j.xpro.2020.100026 PMC7580117

[25] Ju W, Liu K, Ouyang S, Liu Z, He F, Wu J. Changes in N6-methyladenosine modification modulate diabetic cardiomyopathy by reducing myocardial fibrosis and myocyte hypertrophy. Front Cell Dev Biol. 2021;9:702579. 34368154 10.3389/fcell.2021.702579 PMC8334868

[26] Tian C, An Y, Zhao J, Zhu X, Wei W, Ruan G, et al. Bone marrow mesenchymal stem cells reversed ovarian aging-related m6A RNA methylation modification profile in aged granulosa cells. Stem Cell Rev Rep. 2023;19(4):953–67. 36609903 10.1007/s12015-022-10485-y PMC10185602

[27] Yu R, Yu Q, Li Z, Li J, Yang J, Hu Y, et al. Transcriptome-wide map of N6-methyladenosine (m6A) profiling in coronary artery disease (CAD) with clopidogrel resistance. Clin Epigenetics. 2023;15(1):194. 38098098 10.1186/s13148-023-01602-w PMC10722764

[28] Ronaldson-Bouchard K, Ma SP, Yeager K, Chen T, Song L, Sirabella D, et al. Advanced maturation of human cardiac tissue grown from pluripotent stem cells. Nature. 2018;556(7700):239–43. 29618819 10.1038/s41586-018-0016-3 PMC5895513

[29] Sharma A, Garcia G Jr., Wang Y, Plummer JT, Morizono K, Arumugaswami V, et al. Human iPSC-derived cardiomyocytes are susceptible to SARS-CoV-2 infection. Cell Rep Med. 2020;1(4):100052. 32835305 10.1016/j.xcrm.2020.100052 PMC7323681

[30] Toischer K, Rokita AG, Unsold B, Zhu W, Kararigas G, Sossalla S, et al. Differential cardiac remodeling in preload versus afterload. Circulation. 2010;122(10):993–1003. 20733099 10.1161/CIRCULATIONAHA.110.943431 PMC2955196

[31] Baier MJ, Klatt S, Hammer KP, Maier LS, Rokita AG. Ca(2+)/calmodulin-dependent protein kinase II is essential in hyperacute pressure overload. J Mol Cell Cardiol. 2020;138:212–21. 31836540 10.1016/j.yjmcc.2019.12.002

[32] Grossman W, Paulus WJ. Myocardial stress and hypertrophy: a complex interface between biophysics and cardiac remodeling. J Clin Invest. 2013;123(9):3701–3. 23999445 10.1172/JCI69830 PMC3754273

[33] Gheorghiade M, De Luca L, Fonarow GC, Filippatos G, Metra M, Francis GS. Pathophysiologic targets in the early phase of acute heart failure syndromes. Am J Cardiol. 2005;96(6A):11G-G17. 16196154 10.1016/j.amjcard.2005.07.016

[34] Li L, Xu N, Liu J, Chen Z, Liu X, Wang J. M6A methylation in cardiovascular diseases: from mechanisms to therapeutic potential. Front Genet. 2022;13:908976. 35836571 10.3389/fgene.2022.908976 PMC9274458

[35] Zhang B, Jiang H, Wu J, Cai Y, Dong Z, Zhao Y, et al. M6A demethylase FTO attenuates cardiac dysfunction by regulating glucose uptake and glycolysis in mice with pressure overload-induced heart failure. Signal Transduct Target Ther. 2021;6(1):377. 34728610 10.1038/s41392-021-00699-w PMC8563751

[36] Sopic M, Robinson EL, Emanueli C, Srivastava P, Angione C, Gaetano C, et al. Integration of epigenetic regulatory mechanisms in heart failure. Basic Res Cardiol. 2023;118(1):16. 37140699 10.1007/s00395-023-00986-3 PMC10158703

[37] Ke WL, Huang ZW, Peng CL, Ke YP. M(6)A demethylase FTO regulates the apoptosis and inflammation of cardiomyocytes via YAP1 in ischemia-reperfusion injury. Bioengineered. 2022;13(3):5443–52. 35176940 10.1080/21655979.2022.2030572 PMC8974143

[38] Shen W, Li H, Su H, Chen K, Yan J. FTO overexpression inhibits apoptosis of hypoxia/reoxygenation-treated myocardial cells by regulating m6A modification of Mhrt. Mol Cell Biochem. 2021;476(5):2171–9. 33548009 10.1007/s11010-021-04069-6

[39] Xu L, Brink M. mTOR, cardiomyocytes and inflammation in cardiac hypertrophy. Biochim Biophys Acta. 2016;1863(7 Pt B):1894–903. 26775585 10.1016/j.bbamcr.2016.01.003

[40] Zhang D, Contu R, Latronico MV, Zhang J, Rizzi R, Catalucci D, et al. MTORC1 regulates cardiac function and myocyte survival through 4E-BP1 inhibition in mice. J Clin Invest. 2010;120(8):2805–16. 20644257 10.1172/JCI43008 PMC2912201

[41] Zhu Y, Pires KM, Whitehead KJ, Olsen CD, Wayment B, Zhang YC, et al. Mechanistic target of rapamycin (Mtor) is essential for murine embryonic heart development and growth. PLoS One. 2013;8(1):e54221. 23342106 10.1371/journal.pone.0054221 PMC3544830

[42] Buss SJ, Muenz S, Riffel JH, Malekar P, Hagenmueller M, Weiss CS, et al. Beneficial effects of mammalian target of rapamycin inhibition on left ventricular remodeling after myocardial infarction. J Am Coll Cardiol. 2009;54(25):2435–46. 20082935 10.1016/j.jacc.2009.08.031

[43] Flynn JM, O’Leary MN, Zambataro CA, Academia EC, Presley MP, Garrett BJ, et al. Late-life rapamycin treatment reverses age-related heart dysfunction. Aging Cell. 2013;12(5):851–62. 23734717 10.1111/acel.12109 PMC4098908

[44] Volkers M, Konstandin MH, Doroudgar S, Toko H, Quijada P, Din S, et al. Mechanistic target of rapamycin complex 2 protects the heart from ischemic damage. Circulation. 2013;128(19):2132–44. 24008870 10.1161/CIRCULATIONAHA.113.003638 PMC4131547

[45] Wu X, Cao Y, Nie J, Liu H, Lu S, Hu X, et al. Genetic and pharmacological inhibition of Rheb1-mTORC1 signaling exerts cardioprotection against adverse cardiac remodeling in mice. Am J Pathol. 2013;182(6):2005–14. 23567640 10.1016/j.ajpath.2013.02.012

[46] Boutouja F, Stiehm CM, Platta HW. mTOR: a cellular regulator interface in health and disease. Cells. 2019;8(1):18. 30609721 10.3390/cells8010018 PMC6356367

[47] Rajapakse AG, Yepuri G, Carvas JM, Stein S, Matter CM, Scerri I, et al. Hyperactive S6K1 mediates oxidative stress and endothelial dysfunction in aging: inhibition by resveratrol. PLoS One. 2011;6(4):e19237. 21544240 10.1371/journal.pone.0019237 PMC3081344

[48] Marin TM, Keith K, Davies B, Conner DA, Guha P, Kalaitzidis D, et al. Rapamycin reverses hypertrophic cardiomyopathy in a mouse model of LEOPARD syndrome-associated PTPN11 mutation. J Clin Invest. 2011;121(3):1026–43. 21339643 10.1172/JCI44972 PMC3049377

[49] Sciarretta S, Forte M, Frati G, Sadoshima J. The complex network of mTOR signalling in the heart. Cardiovasc Res. 2022;118(2):424–39. 33512477 10.1093/cvr/cvab033 PMC8932297

[50] Takagi Y, Kobayashi T, Shiono M, Wang L, Piao X, Sun G, et al. Interaction of folliculin (Birt-Hogg-Dube gene product) with a novel Fnip1-like (FnipL/Fnip2) protein. Oncogene. 2008;27(40):5339–47. 18663353 10.1038/onc.2008.261

[51] Wang L, Harris TE, Roth RA, Lawrence JC Jr. PRAS40 regulates mTORC1 kinase activity by functioning as a direct inhibitor of substrate binding. J Biol Chem. 2007;282(27):20036–44. 17510057 10.1074/jbc.M702376200

[52] Feng S, Qiu G, Yang L, Feng L, Fan X, Ren F, et al. Omeprazole improves chemosensitivity of gastric cancer cells by m6A demethylase FTO-mediated activation of mTORC1 and DDIT3 up-regulation. Biosci Rep. 2021;41(1):BSR20200842. 10.1042/BSR20200842 PMC7843496 33393595

[53] Wang F, Liao Y, Zhang M, Zhu Y, Wang W, Cai H, et al. N6-methyladenosine demethyltransferase FTO-mediated autophagy in malignant development of oral squamous cell carcinoma. Oncogene. 2021;40(22):3885–98. 33972683 10.1038/s41388-021-01820-7

[54] Zhao L, Kong X, Zhong W, Wang Y, Li P. FTO accelerates ovarian cancer cell growth by promoting proliferation, inhibiting apoptosis, and activating autophagy. Pathol Res Pract. 2020;216(9):153042. 32825930 10.1016/j.prp.2020.153042

[55] Jin S, Zhang X, Miao Y, Liang P, Zhu K, She Y, et al. M(6)A RNA modification controls autophagy through upregulating ULK1 protein abundance. Cell Res. 2018;28(9):955–7. 30046135 10.1038/s41422-018-0069-8 PMC6123428

[56] Wang X, Wu R, Liu Y, Zhao Y, Bi Z, Yao Y, et al. M(6)A mRNA methylation controls autophagy and adipogenesis by targeting Atg5 and Atg7. Autophagy. 2020;16(7):1221–35. 31451060 10.1080/15548627.2019.1659617 PMC7469583

[57] Chen X, Wang J, Tahir M, Zhang F, Ran Y, Liu Z, et al. Current insights into the implications of m6A RNA methylation and autophagy interaction in human diseases. Cell Biosci. 2021;11(1):147. 34315538 10.1186/s13578-021-00661-x PMC8314498

[58] Settembre C, Di Malta C, Polito VA, Garcia Arencibia M, Vetrini F, Erdin S, et al. TFEB links autophagy to lysosomal biogenesis. Science. 2011;332(6036):1429–33. 21617040 10.1126/science.1204592 PMC3638014

[59] Hollebecque A, Houede N, Cohen EE, Massard C, Italiano A, Westwood P, et al. A phase Ib trial of LY2584702 tosylate, a p70 S6 inhibitor, in combination with erlotinib or everolimus in patients with solid tumours. Eur J Cancer. 2014;50(5):876–84. 24456794 10.1016/j.ejca.2013.12.006

[60] de la Pena JB, Kunder N, Lou TF, Chase R, Stanowick A, Barragan-Iglesias P, et al. A role for translational regulation by S6 kinase and a downstream target in inflammatory pain. Br J Pharmacol. 2021;178(23):4675–90. 34355805 10.1111/bph.15646 PMC9169231

[61] Grimshaw KM, Hunter LJ, Yap TA, Heaton SP, Walton MI, Woodhead SJ, et al. AT7867 is a potent and oral inhibitor of AKT and p70 S6 kinase that induces pharmacodynamic changes and inhibits human tumor xenograft growth. Mol Cancer Ther. 2010;9(5):1100–10. 20423992 10.1158/1535-7163.MCT-09-0986 PMC4825853

[62] Artemenko M, Zhong SSW, To SKY, Wong AST. P70 S6 kinase as a therapeutic target in cancers: more than just an mTOR effector. Cancer Lett. 2022;535:215593. 35176419 10.1016/j.canlet.2022.215593

