# Supplementary material for: Deregulation of m6A-RNA methylation impairs adaptive hypertrophic response and drives maladaptation via mTORC1-S6K1-hyperactivation and autophagy impairment
Source: Cell Commun Signal. 2025 Dec 2;23:522. doi: 10.1186/s12964-025-02509-0 (PMC12690826; doi:10.1186/s12964-025-02509-0)
Supplement: Supplementary file 1 — Supplementary Material 1 [file 12964_2025_2509_MOESM1_ESM.pdf]

Supplementary figure 1.

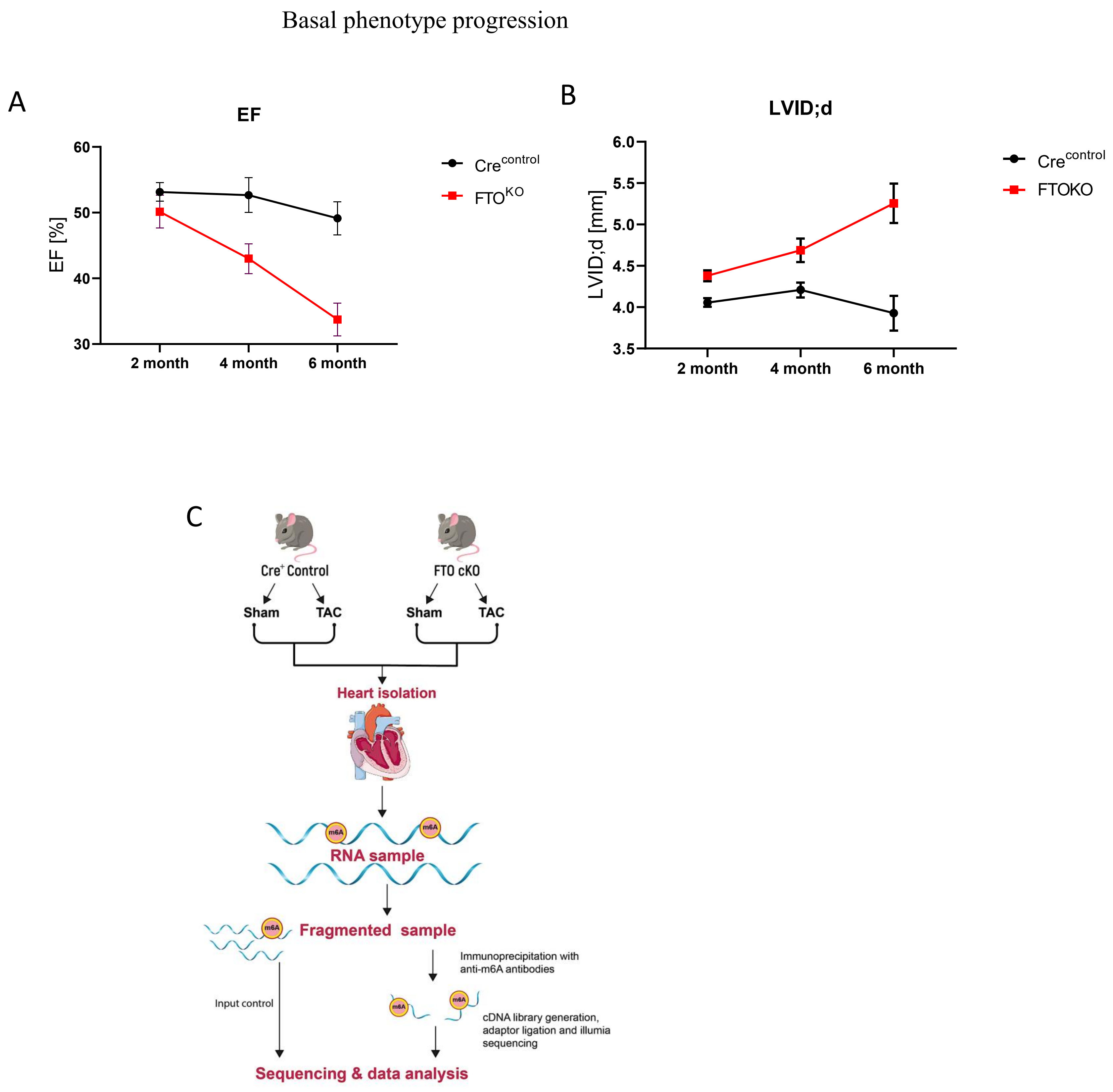

Echocardiographic analysis for FTOcKO animals at different timepoints (2,4 and 6 months); A) EF (ejection fraction) and B) LVID,d (Left Ventricular interdimensional end diastolic diamenter) is shown for control (CreC and FTOcKO mice); C) Schematic representation of methylated RNA immunoprecipitation (MeRIP) sequencing

Supplementary figure 2.

Transcriptomics of FTOcKO mice vs. Cre Control

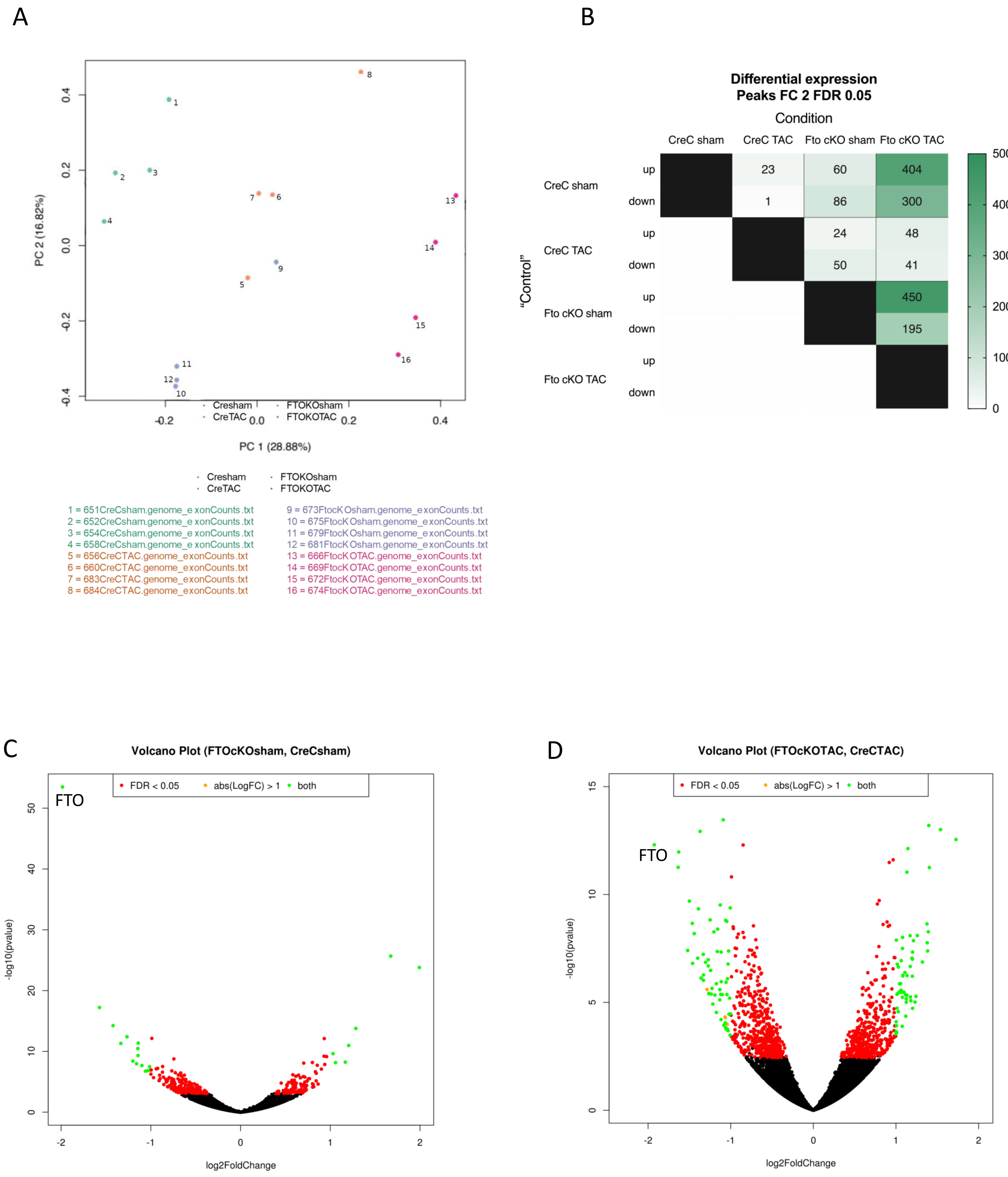

Transcriptomics of FTOcKO and Cre Control animals: A) PCA plots of the animals groups CreC Sham, CreC TAC, FTOcKO Sham and FTOcKO TAC; B) The number of differential expressed genes for each condition (CreC sham, CreC TAC, FTOcKO sham, FTOcKO TAC) is represented in a table format; C) Volcano plot for the condition FTOcKO sham vs. CreC sham; D) Volcano plot (FTOcKO TAC vs. CreC TAC).

Supplementary figure 3.

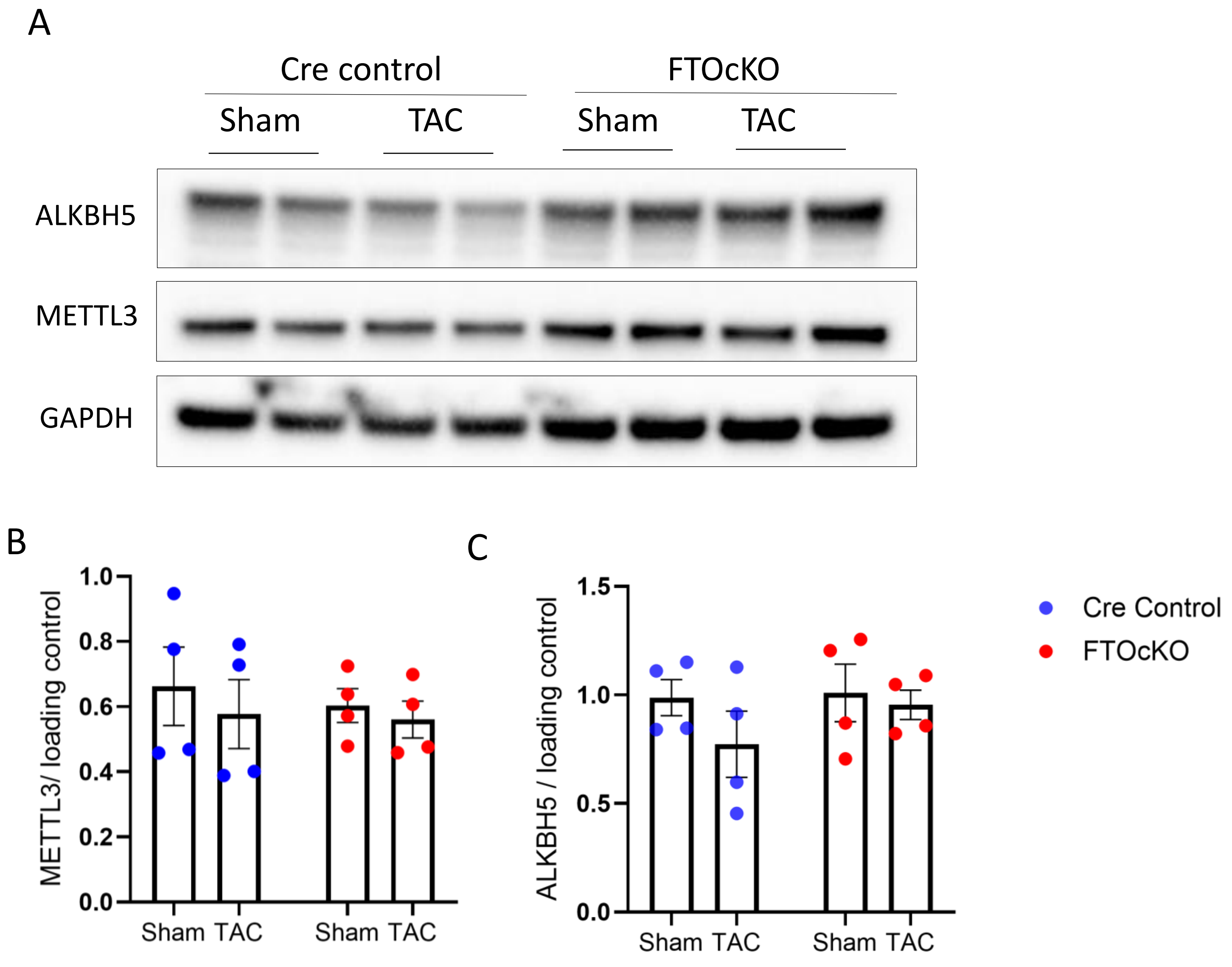

A) Representative western blot data for ALKBH5 and METTL3 protein levels in Cre C and FTOcKO animals (both sham and TAC); B) and C) shows the pooled quantitative densitometry analysis for METTL3 and ALKBH5 respectively. Statistics by ordinary two-way ANOVA with multiple comparisons, no significance detected.

Supplementary figure 4.

A

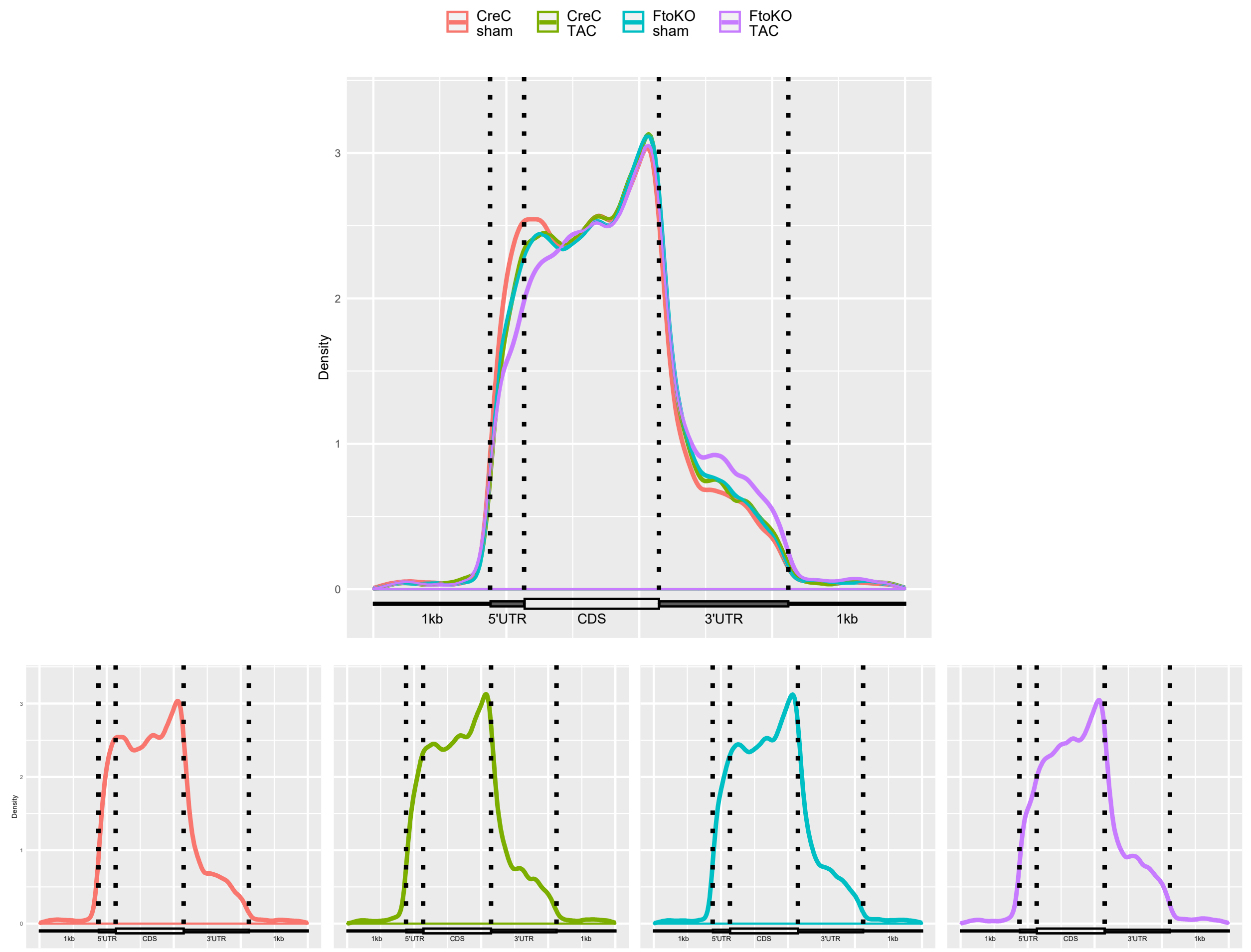

B

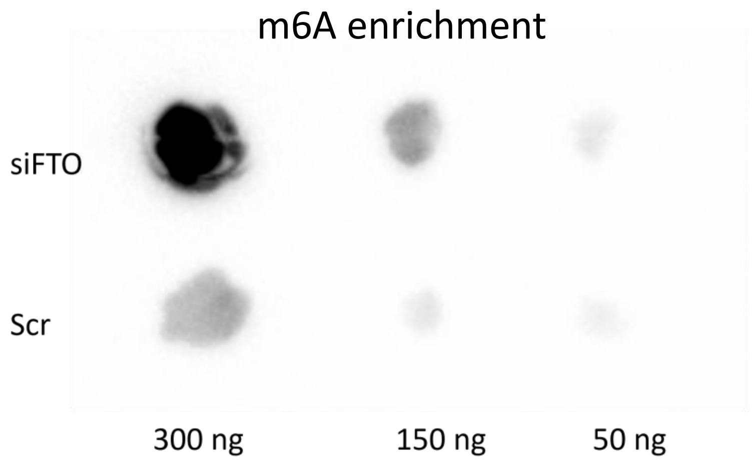

C

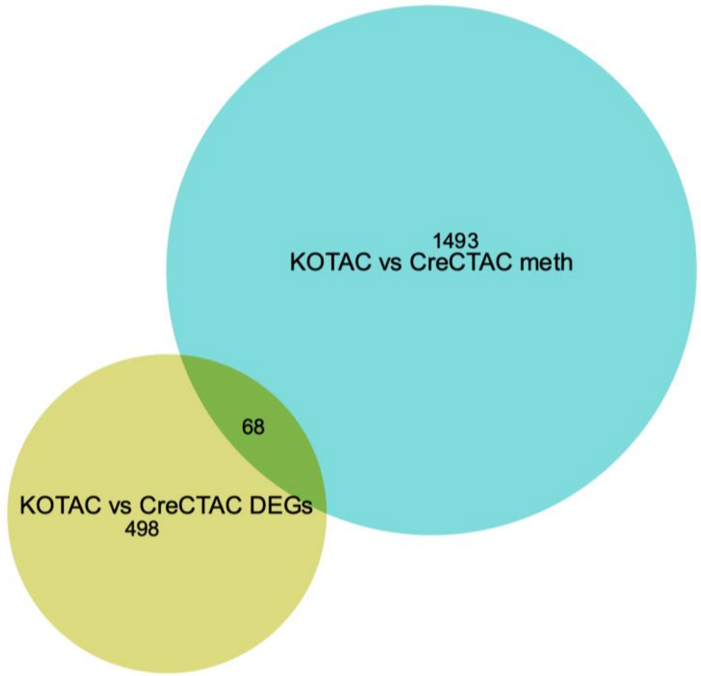

MeRIP seq data :

A) Data of m6A peak distribution of MeRIP samples of heart tissues of FTOcKO and Cre Control groups that underwent sham and TAC surgery.

B) Dot blot assay was performed with the total mRNA extracted from Scr and FTO knockdown hiPS-CMs. Increased hypermethylation detected upon FTO loss in the cardiomyocytes.

C) Comparison of differential methylation/differential expression in FTOcKO TAC in relation to Cre Control TAC. FC>=1.2 in methylation ; FC >= 1.5 in expression;

Supplementary figure 5.

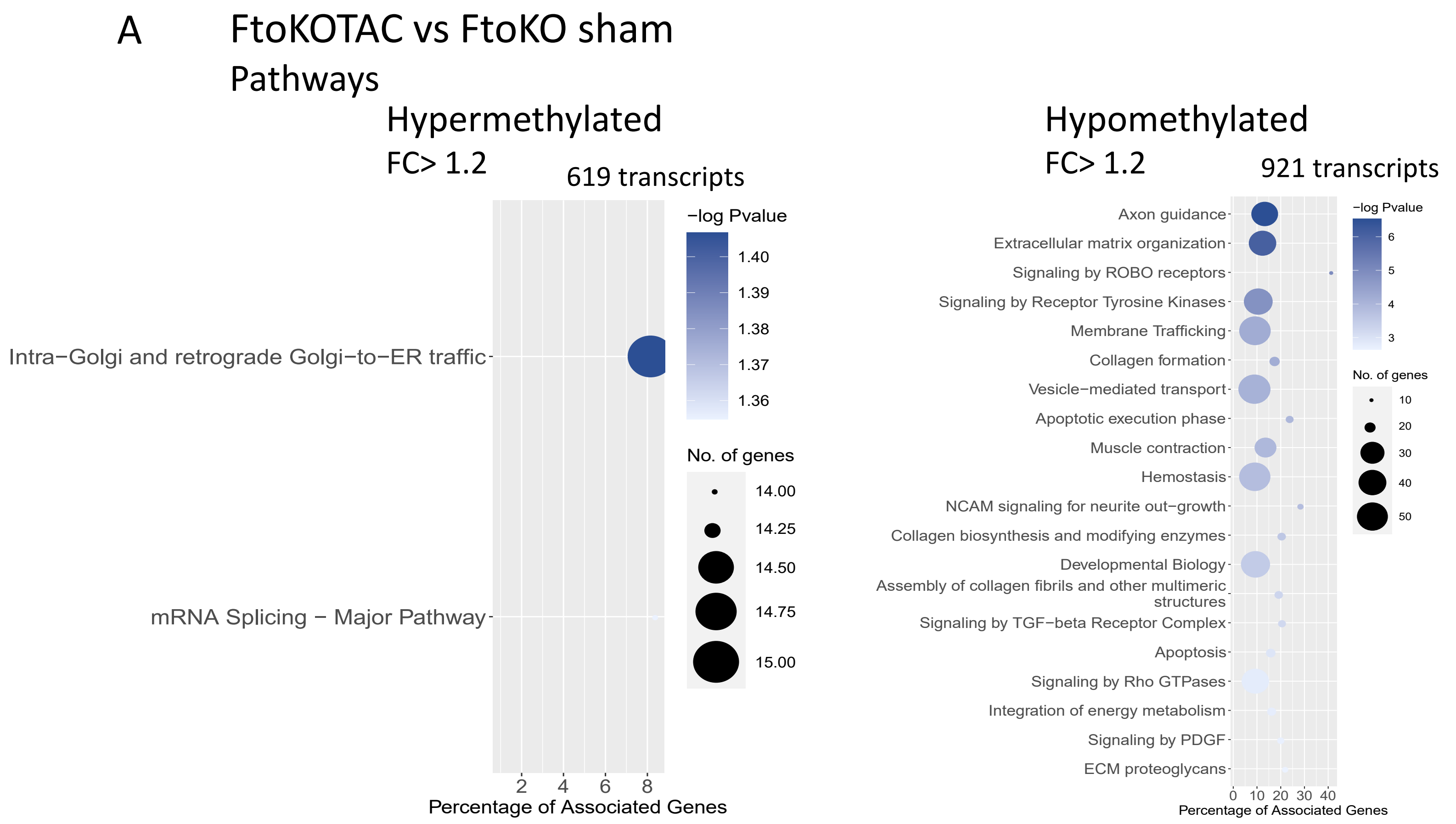

A) Gene Ontology enrichment of differentially methylated transcripts, FTOcKO TAC vs. FTOcKO sham, with FC >1.2 FDR 0.05 : the biological process of differentially hypermethylated transcripts and differentially hypomethylated transcripts are shown.

Supplementary figure 6.

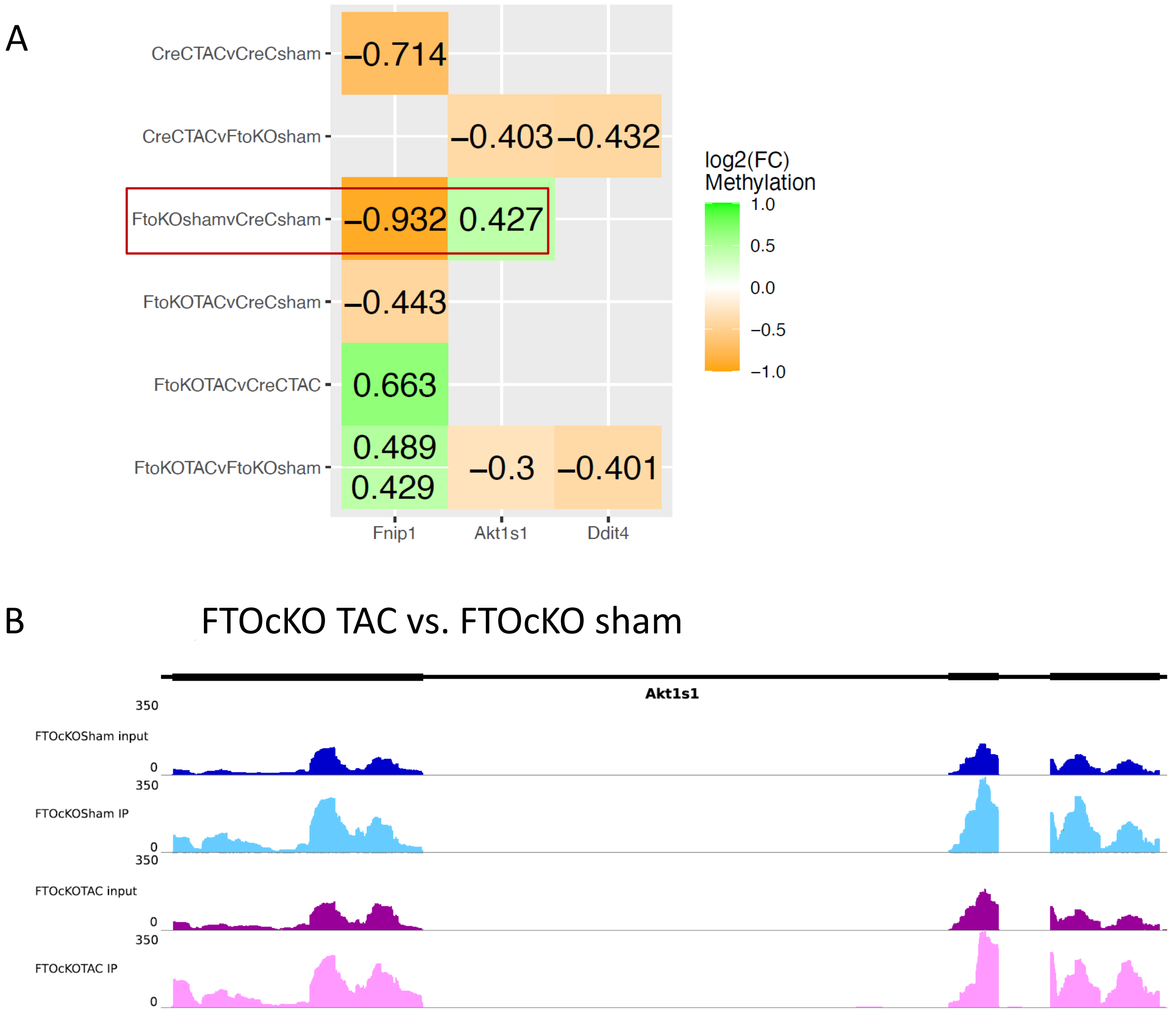

A) The above table represents that log2 (FC) methylation values for the transcripts of mTORC1 pathway namely, Fnip1, Akt1s1 and Ddit4. There is an increased hypomethylation in the transcripts of Fnip1 and hypermethylation in the transcripts of Akt1s1 (regulator of mTORC1) in FTOcKO sham compared to the CreC sham.

The transcript level m6A methylation map of AKT1S1 under specific exon regions are shown in B) FTOcKO TAC vs FTOcKO sham.

Supplementary figure 7.

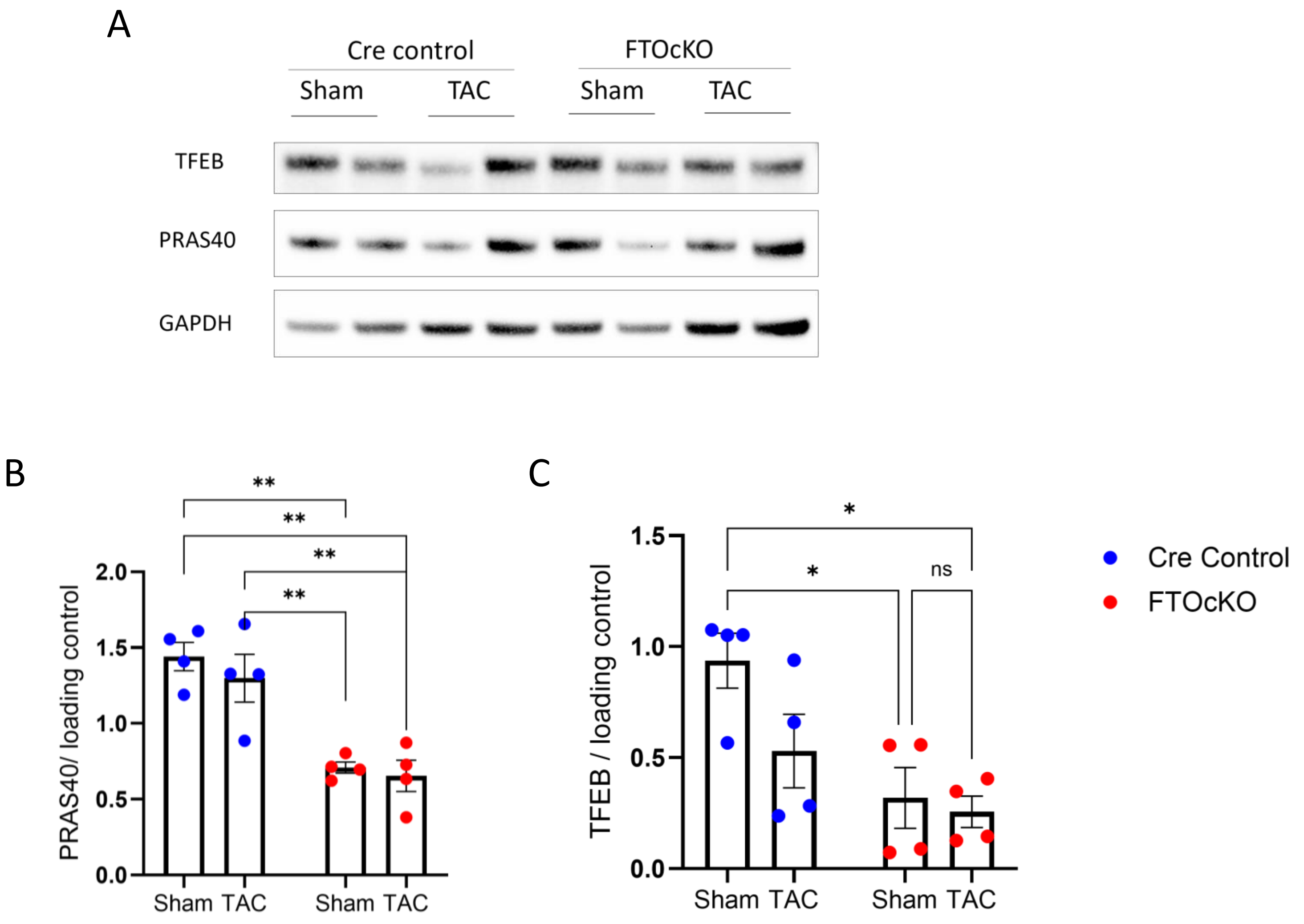

A) Representative western blot data for TFEB and PRAS40 protein levels in Cre C and FTOcKO animals (both sham and TAC); B) and C) shows the pooled quantitative densitometry analysis for PRAS40 and TFEB respectively. Statistics by ordinary two-way ANOVA with multiple comparisons, **$p<0.005$; *$p<0.05$.

Supplementary figure 8.

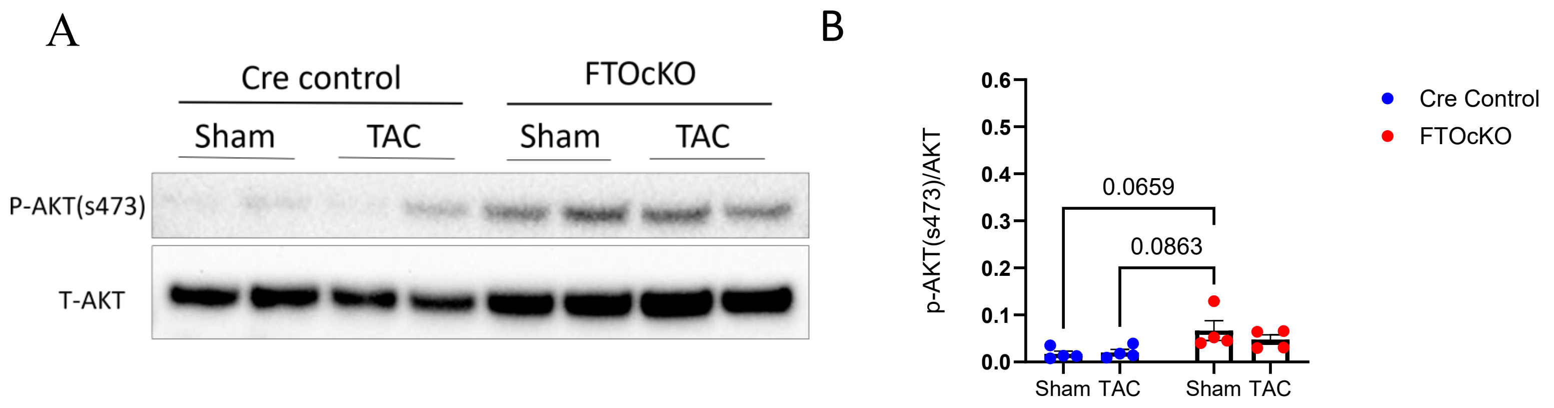

A) western blot data of representing p-AKT(ser473) activity in Cre C and FTOcKO animals (both sham and TAC); B) shows the pooled quantitative densitometry analysis for p-AKT(s473) normalized with total AKT. Statistical analysis performed using ordinary two-way ANOVA with multiple comparisons.

Supplementary figure 9.

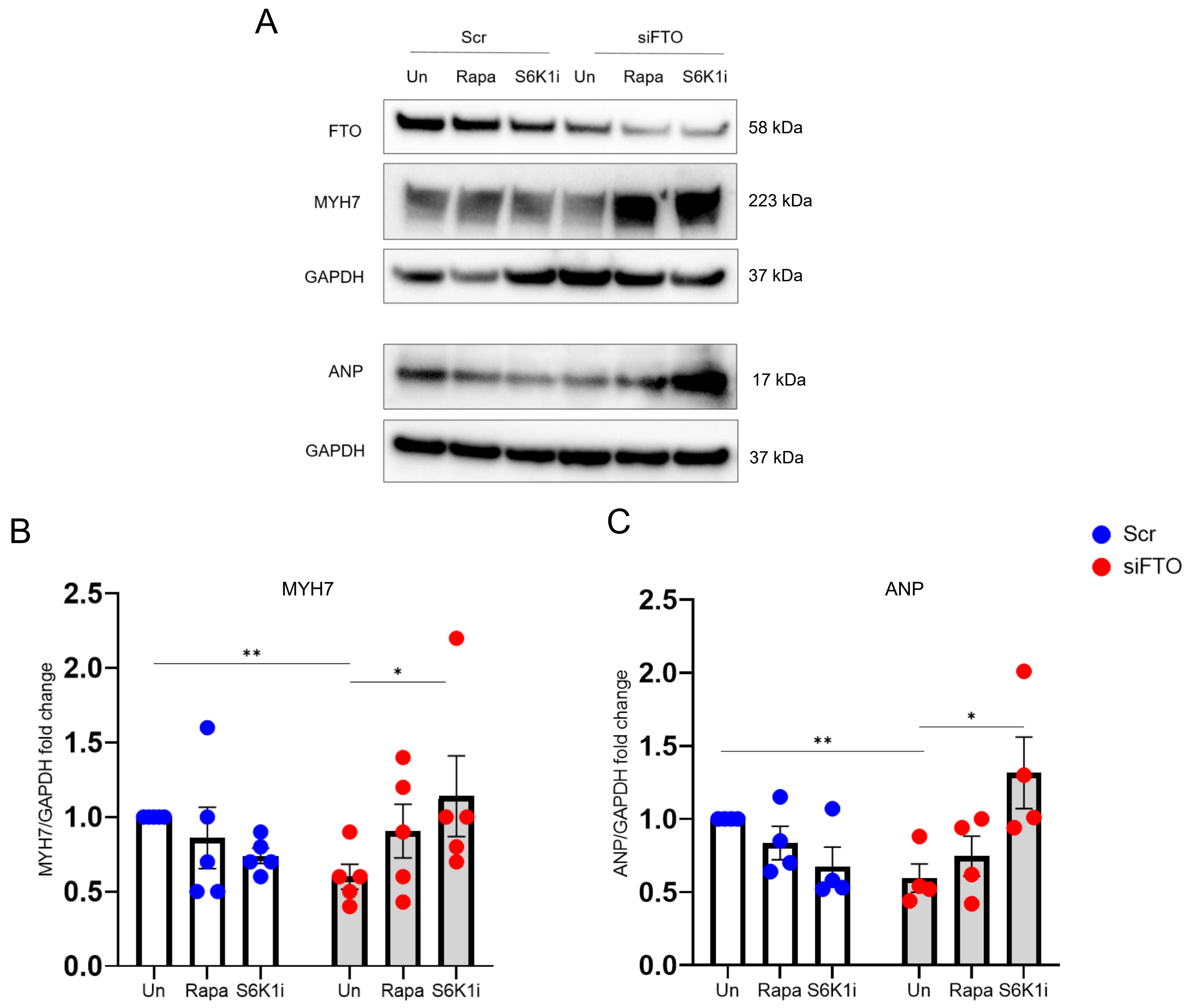

The hiPS-CMs were transfected with siRNAs of FTO and scr for 24 hrs, further treated with mTORC1 inhibitors either Rapamycin (Rapa) or PF-4709671(S6Ki) with 50 nM and 5 µM final concentration respectively, for another 24 hrs: A) representative western blots showing the hypertrophic markers MYH7 and ANP ; B) pooled densitometry analysis for MYH7 ; C) pooled densitometry measurement for ANP; *p<0.05 ; **p<0.01 by using student's t-test.
