# Supplementary material for: Deregulation of m6A-RNA methylation impairs adaptive hypertrophic response and drives maladaptation via mTORC1-S6K1-hyperactivation and autophagy impairment
Source: Cell Commun Signal. 2025 Dec 2;23:522. doi: 10.1186/s12964-025-02509-0 (PMC12690826; doi:10.1186/s12964-025-02509-0)
Supplement: Supplementary file 2 — Supplementary Material 2 [file 12964_2025_2509_MOESM2_ESM.docx]

**SUPPLEMENTARY METHODS S1**

1. **Mice**

Cardiomyocyte specific FTO knockout (FTOcKO) mice were generated by mating male FTO^fl/fl^ mice (Exon 3 of *Fto* is flanked by loxP sites, was available inhouse) with the cardiomyocyte specific *αMHC-Cre* females (*αMHC-Cre*, Jackson no. 011038, C57BL/6N and C57BL/6J mixed background [Agah et al. 1997]). And for the experiments, *αMHC-Cre*^+^;*FTO* ^fl/fl^ mice (Cre+ fl/fl) bearing the homozygous *Fto* knock-out were used and named as FTOcKO mice. Further *αMHC-Cre*^+^ mice (Cre+ wt/wt) were used as wildtype control to rule out Cre- recombinase and loxP system derived effects.

1. **Transverse aortic constriction (TAC) in mice**

All the experimental investigations conforms to the Guide for the Care and Use of Laboratory animals (NIH publication No. 85–23, revised 1996) and was implemented in accordance with the ethical standards laid down in the [Declaration of Helsinki](http://link.springer.com/search?dc.title=Declaration+of+Helsinki&facet-content-type=ReferenceWorkEntry&sortOrder=relevance) 1964.

Surgery was carried with a minimally invasive approach. 8-week-old mice were randomly selected for both Sham and TAC surgery. Initially, the animals were anaesthetized using intraperitoneal injections with mixture of Medetomidin (0.5mg/kg), Midazolam (5mg/kg) and Fentanyl (0.05 mg/kg). A 26 gauge needle was tied against the aorta using a 5-0 Polyviolene (non-absorbable) suture for TAC surgery, and Sham animals underwent the similar procedure without banding of the transverse aorta.

1. **Echocardiography**

Transthoracic echocardiography using Vevo 2100 system (Visualsonics) were examined for mice at different age groups (2, 4 and 6 months), and also for the mice that underwent TAC or Sham surgery (1 week post-surgery). The animals were anaesthetized using 1.5% isoflurane, and the heart rate, respiratory rate and body temperature were monitored. 2D guided M-mode images were recorded in both long axis and short axis with the help of of an MS-400 30 Mhz transducer (Visualsonics) (Pistner et al. 2010). For the analysis of the recorded images, VevoLab software were applied (Version 3.1.0, Visualsonics). The examiner were blinded for the assigned animal groups.

1. **Murine LV isolation**

For the heart isolation, the mice were anesthetized with isoflurane and sacrificed by cervical dislocation. The thorax were opened and the exposed heart were then cut above the aorta and washed in sterile NaCl solution. Further, the heart was perfused with sterile NaCl by inserting a 21 gauge blunt needle into the aorta. The heart was weighed, and the LV snap alone were separated and frozen in liquid nitrogen for the experimental investigations. The tibia was also isolated from the muscle tissue and its length was measured in order to calculate heart weight/ tibia length ratio.

1. **Methylated RNA immunoprecipitation (MeRIP) and analysis**

Initially, total RNA were isolated from the left ventricles with Tirzol reagent (Thermo Fisher Scientific, #15596018) as per the manufacturer's instruction. The isolated RNA were purified by DNAse treatment, and further rRNA was removed by using NEBNext rRNA Depletion Kit (NEB, #E6310, Ipswich, MA). The purified RNA was fragmented to about 80 nt by RNA fragmentation reagent at 70°C for 12 minutes(#AM8740, Thermo Fisher Scientific, Waltham, MA). A small part of RNA (500ng) were considered as input and the rest of the fragmented RNA were used for immunoprecipitation with anti-m6A polyclonal antibody (synaptic systems, #202003). The sample RNA was initially diluted in10mM Tris-HCl pH 7.4, 150mM NaCl and 0.1% (vol/vol) Igepal CA-630 buffer supplemented with 200 U RNasin Plus RNase Inhibitor (#2611, Promega) and 2mM RVC (#R3380, Sigma-Aldrich). 5ug of anti-m6A polyclonal antibody conjugated to Dynabeads Protein G (Thermo Fisher Scientific, #10003D) was added to the RNA samples and was incubated at 4°C ON. In the following day, the beads were washed with 1x IP buffer three times, and the m6A methylated RNA was eluted twice with 6.7mM N6-methyladenosine (Sigma-Aldrich, M2780) in 1x IP buffer. Further, the eluted RNA were purified using ZymoResearch RNA Clean and Concentrator-5 (Zymo Research, #R1015). The RNA samples were processed for cDNA library preparation using TruSeq stranded Total RNA Library Prep Kit (#20020596, Illumina) and subjected to sequencing with Illumina HiSeq 2000. The generated reads were mapped and the peaks showing significant enrichment for m6A in comparison to the input samples were detected with the MeTPeak package.

1. Methylated RNA immunoprecipitation (MeRIP)- qPCR

For the validation of MeRIP-seq data, the m6A enrichment of the transcripts AKT1S1 and TFEB were analysed by performing MeRIP from the Scr and FTO silenced hiPS-CMs using Magna-MeRIP^TM^ m6A Kit (catalog no. 17-10499). MeRIP was carried as per the manual`s instruction. The eluted samples of m6A immunoprecipitation and IgG (control) RIP, were subjected to cDNA synthesis using Quantitect Reverse Transcription Kit Catalog no#205313.

Further, cDNAs of m6A RIP and IgG RIP are subjected to real time PCR using the specific taqman pre-mixed primers for AKT1S1 (TaqMan Gene expression Assay ID   Hs00982883_m1 ; AKT1S1); TFEB (TaqManTM Gene Expression Assay Hs00292981_m1; TFEB) from Invitrogen. The analysis for m6A enrichment is performed by using Δ ΔCt method, where the ΔCt for m6A and the negative control (IgG) is normalized with their respective inputs and further Δ ΔCt is obtained by subtracting by ΔCt of m6A with ΔCt of IgG (negative). And the fold enrichment is calculated with 2^(- Δ ΔCt). The fold enrichment is plotted in the graph for each transcript.

1. Real time PCR

The total RNA extracted from Scr and siFTO hiPS-CMs were subjected to cDNA synthesis using Quantitect Reverse Transcription Kit (Cat. No. #205313) and the corresponding cDNAs were considered for Real time PCR. The Taqman Universal PCR master mix (Cat. No. 4304437) and the specific qPCR primers for AKT1S1 (TaqMan Gene expression Assay ID   Hs00982883_m1 ; AKT1S1); TFEB (TaqManTM Gene Expression Assay Hs00292981_m1; TFEB); and FTO (Taqman Gene expression Assay ID Hs01057143_m1 ; FTO) were purchased from Invitrogen.

1. **Human induced pluripotent stemcells (hiPSC)**

For this sutdy, the human patient derived pluripotent stemcells (hiPSCs) were kindly provided by Dr. Katrin Streckfuß-Bömeke (Institue for pharmacology and toxicology, University of Würzburg, Germany) and PD Dr. Antje Ebert (University Medical Center Göttingen, Department of Cardiology and Pneumology).

1. **hiPS cells differentiation into cardiomyocytes (hiPS-CMs) and culturing**

hiPS cells were differentiated into beating ventricular cardiomyocytes with the help of an adapted protocol from Kleinsorge and Cyganek et al. (Kleinsorge and Cyganek 2020).

1. **siRNAs and inhibitors**

The siRNAs were purchased from Qiagen. For FTO silencing, the product HS_FTO flexitube siRNA with Geneglobe ID-S104177530 (catalog #1027417) were considered. All stars negative control siRNA (catalog #1027280) were considered as Scr control.

For induction of stress and hypertrophic responses in iPS-CMs, Endothelin-1 (ET-1) (Sigma.#E7764) (Archer et al. 2017) were used.

For mTORC1-S6K1 inhibition, the inhbitors 1. Rapamyin (#553210-10MG) and 2. S6K1 inhibitor PF-4708671 (#559273-10MG) were purchased from Sigma Aldrich Chemie GmbH.

For late-autophagy inhibition, the inhibitors Bafilomycin A1 (CAS 88899-55-2) and Chloroquine were considered.

1. **Transfections in hiPS-CMs**

The hiPS-CMs cultured in the 6 well plates/12 well plates were transfected with siRNAs using Hiperfect Transfection reagent (Qiagen; #301705). 50 nM and 100 nM siRNAs for 12 well and 6 well plates respectively were considered for the transfection in hiPS-CMs.

1. **Antibodies for Western blot and Immunofluorescence (IF)**

The antibodies used for western blot are all diluted in the ratio 1:1000 except for the house keeping genes (1:10,000). The order details of the antibodies are ANP(#ab126149), MYH7 (MA5-32986), FTO (#NBP2-29512), cleaved Caspase3 (#9664S), cleaved PARP (#9544S,#9541S), P-S6 Ribosomal protein (S235/236) (#4858S), P-p70 S6 kinase (T389) (#9234S), P-4E-BP1(T37/46) (#2855S), total S6 Ribosomal protein (#2217S), p70-S6Kinase(2708S), 4E-BP1(#9644T), SQSTM1/p62 (#5114S), LC3B(#2775S), Actin(#4967) and GAPDH (#2118S) , PRAS40 (#2610S), TFEB (#4240S), METTL3(#96391S), ALKBH5(#80283S), p-AKT(s473) (#9271S), AKT (#9272S).

For immunofluroscence the antibodies were diluted in the ratio 1:100 : alpha-actinin(sarcomeric) (#A7732), phospho-S6(ser235,ser236) (#MA5-15140), p62 (#GP62-C-WBC), LC3B (#2775S), anti-mouse IgG secondary antibody Alexafluor plus488 (#A32723), anti-rabbit IgG(H+L) cross adsorbed secondary antibody Alexafluor 555 (#10082602) , FITC-affinipure anti-rabbit IgG (H+L) (#111-095-003), Cy3- conjugated affinipure anti-Guinea Pig IgG (H+L) (#706-165-148).

1. **Western blot analysis**

Protein samples were prepared with a total concentration of 30µg and a volume of 30µl was loaded in the gel for SDS-PAGE. The samples were incubated at 95°C for 10 minutes and loaded on 4-12% precast TRIS gels for electrophoresis. 5µl of the pre-stained standard marker was loaded. 1mL of antioxidant was added to prevent heating of the gels during electrophoresis. Electrophoresis was performed at 200V for 50 minutes. Once electrophoresis was finished, the gels were placed in transfer buffer until transfer. The transfer buffer consisting of methanol and 20X transfer buffer should always be cold until use. The transfer is performed using PVDF membranes which is activated in methanol and equilibrated in transfer buffer prior to the transfer. The transfer was performed at 30V for 1 hour and 40 minutes. After the transfer, the membranes were blocked 2.5% BSA and dry milk powder in TBST for 1 hour and 12 rpm. The membranes were later incubated overnight in the cold room with a primary antibody diluted in a ratio of 1:1000 in 5% BSA with sodium azide. On the next day, the membranes were washed with TBST and incubated for 1 hour with a secondary mouse or rat antibody diluted in a ratio of 1:10000. The membranes are then developed using a BioRad development camera.

1. **Histology**

Hearts were harvested from the mice and were fixed in 4% formalin, further embedded with paraffin and sectioned (6µm). Those LV sections were considered for Immunohistochemistry and Immunofluorescence.

The sections were stained with fluorescein-conjugated **wheat germ agglutinin** (WGA-Alexa Fluor 594; Invitrogen) for the assessment of cross-sectional area using ImageJ software (NIH; Bethesda).

Further, the LV sections were stained for Fibrosis by using **Picro Sirius Red Stain Kit (Connective Tissue Stain) #ab150681**; and the fibrotic area is measured using ImageJ software NIH; Bethesda). And the % fibrosis is plotted in prism graph.

1. **TUNEL assay (for LV sections and for iPS-CMs)**

The LV sections were de-paraffinized, permeablized and the slides were labelled with TUNEL working solution (In situ Cell Death Kit, TMR red #12156792910) or TUNEL Staining (#11684795910 - In Situ Cell Death Detection Kit, Fluorescein-Roche) and incubated at 37°C for 1 hour.

The TUNEL assay was performed in the iPS-CMs by initially coating the cardiomyocytes in the coverslips (which were plated in the 12 well plates for the transfection and treatments). Further, the cells were incubated overnight with primary antibody (alpha-actinin), the following day, the sections were incubated with respective secondary antibody (Alexa Fluor plus 488) for an hour at RT. The sections were finally washed with PBS before mounting with DAPI and proceeded with fluorescent microscopy analysis.

1. **P-S6 staining (for LV sections and for hiPS-CMs)**

Phospho-S6 staining was performed for the LV sections using the enhancer Tyramide Signal Amplication (TSA) kit ( Alexa Fluor 488 Tyramide Super Bosst Kit # B40922).

Phospho-S6 staining were performed in hiPS-CMs by plating the cells in coverslips of 12 well plates (transfections were performed in 12 well plates). The fixed and permealized cells were incubated with primary antibodies (alpha-actinin and phospho-S6(ser235,ser236)) overnight at 4°C. The following day, the cells were incubated with their respective secondary antibody solutions at RT for 1 hour. The cells were mounted with DAPI for microscopic analysis.

1. **P62 and LC3 localization (for hiPS-CMs)**

The CMs were double stained (as mentioned in 13.2.) Here, the cells were incubated with primary antibodies (SQSTM1/p62 and LC3 B) overnight at 4°C. And the following day, the cells were washed and incubated with their respective secondary antibodies (FITC anti-rabbit and Cy3 anti-GP as mentioned in section 10) and the stained coverslips were mounted with DAPI and headed for microscopic analysis.

1. **Tandem mRFP-GFP-LC3 assay (for hiPS-CMs)**

The CMs (approximately 350 cells per well) were plated in 24 well plates, the cells were initially transduced with tandem sensor RFP-GFP-LC3B (catalog no. P36239), by adding 2 ul of the sensor to the cells and incubated for 24 hours, further, transfection is performed with Scr and FTO siRNAs and subsequently treated with or without Bafilomycin A1 for 12 hours. The cells are then exposed to Live cell imaging for the microscopical analysis.

1. **Dot blot assay for m6A enrichment (for hiPS-CMs)**

The total mRNA of Scr and siFTO-hiPS-CMs, were initially denatured at 95°C for 5 minutes to disrupt the secondary structure. Further, chilled on ice immediately for 5 mins and spotted on activated PVDF membrane. The crosslinking of mRNA to the membrane is carried by UV light exposure for 5 mins. Further, the membrane is blocked and incubated with primary antibody buffer overnight. The m6A antibody is purchased from Cell Signaling technology with cat. No. 56593S. The m6A spots are developed on the next day after incubating with corresponding secondary antibody solution.

1. **Statistical analysis**

Statistical analysis of the echocardiographic data was performed in the GraphPad Prism Software (v. 8.4.2). Ordinary two-way ANOVA and Tukey`s multiple comparison analysis was performed for Echocardiographic analysis, WGA staining, Fibrosis and Densitometry analysis for mice groups (Sham/TAC and cKO/Control). Densitometry analysis of the protein bands of western blots for scr and siFTO-hiPS-CMs were examined by 1-sample t-tests (using Graphpad prism 9.5.1). The Results were normalized to their respective control conditions and the ratios in which a normal distribution of results cannot be proven, were analyzed. P value <0.05 was considered as statistically significant.
